# Supplementary material for: Evidence for the first globular cluster stellar stream beyond the Milky Way
Source: Nature. 2026 Aug 12;656(8129):843–7. doi: 10.1038/s41586-026-10878-w (PMC13518218; doi:10.1038/s41586-026-10878-w)
Supplement: Supplementary file 1 [file 41586_2026_10878_MOESM1_ESM.pdf]

nature portfolio

Peer Review File

Evidence for the First Globular Cluster Stellar Stream beyond the Milky Way

Corresponding Author: Ms Julie Holm

Any redactions in this file are there to maintain patient confidentiality, the confidentiality of unpublished data, or to remove third-party material.

This file contains all reviewer reports in order by version, followed by all author rebuttals in order by version.

Attachments originally included by the reviewers as part of their assessment can be found at the end of this file.

Version 1:

Reviewer comments:

Referee #1

(Remarks to the Author)
Summary & Significance: This paper presents the discovery of globular cluster stream around a dwarf galaxy located 35 Mpc from the Milky Way, as well as a dynamical constraint on the halo mass of the host ultra-diffuse galaxy. Both of these results have significant implications for the fields of galaxy evolution and the astrophysical study of dark matter, and therefore are of clear interest to a broad scientific community. I have some suggestions for additional analysis and discussion, described below. After these comments are addressed, I recommend the publication of this paper in Nature.

Interpretation & Conclusions: My primary comments are related to the question of whether the stream is the remnant of a disrupted globular cluster or dwarf galaxy. The discovery of a globular cluster stream would be novel and deserving of publication in Nature, while the discovery of a dwarf galaxy stream (of which many are known) would not. Overall, I find the analysis convincing, but I have requests for clarification and suggestions for additional tests to further strengthen the conclusion that this is in fact a globular cluster stellar stream. Generally, I think it is important to show that the stream is not consistent with a dwarf galaxy origin, in addition to showing that it is consistent with a globular cluster origin.

Analysis & Statistics:
- The measurement of the width of the stream is a key piece of evidence for its globular cluster origin. Since the surface brightness of the stream decreases at larger distances from the central track, it would be good to perform additional tests to ensure that detection limits are not leading to an artificial decrease in the measured width. I suggest injecting simulated streams of different widths into the data and testing your ability to recover the correct width. As mentioned above, I think it is important to demonstrate that the data is not also consistent with a larger width.

- In addition, it could be helpful to include additional information about the confidence of the measured properties of the globular cluster progenitor. For example, how does the inferred size and luminosity depend on the selection region?

- The significance of the similarity in the colors between the stream and the GC candidates would be easier to interpret with some qualitative discussion of the probability of the colors of the candidate stream and GC matching in the case that they are not associated.

- This sentence is not clear to me: “Pal 5 contains 3000 stars in the DESI g band between magnitudes 23 and 20, which we use as a threshold for our stellar sample.” Do you mean that you use that sample of stars, combined with an IMF, to determine the total luminosity of the stream beyond these magnitude limits?

- How does the level of disruption (i.e. the length of the stream) affect the estimated surface brightness? A stream that is less massive and less disrupted could have the same surface brightness as a stream that is more massive and more disrupted. The decision to assume that the disruption level is similar to Pal 5 should be more clearly motivated, or a broader range of stream lengths should be accounted for in the calculation.

- When sampling from an isochrone to generate a stellar population, are the age and metallicity selected to match Pal 5? The paper mentions that the candidate stream is consistent with being younger and more metal rich (and therefore brighter) than Pal 5. Why not sample from a broader range of isochrones to test the detectability of streams across the range of stellar population parameters that are consistent with the measured color?

- In addition to using Pal 5 as a comparison to calculate surface brightness, it would be interesting to use the stream from the dynamical model. I suggest first doing a rough surface brightness calculation along the stream, to determine whether the stream would be detectable given the expected mass and level of disruption, and second whether a larger extent of the stream is expected to be observable (does the surface brightness of the tail drop off quickly enough? If not, is there a proposed explanation?). You could also consider injecting the simulated stream into the actual image to similarly test whether the observed morphology would match the data. Of course, all of this should be presented with clear caveats, and I would not expect the simulated stream to match perfectly, but I think it would help strengthen the interpretation.

- By more robustly estimating the detectability of the simulated streams, you may be able to make some additional statements about the properties of the GC progenitor (e.g. if you are able to estimate the length of the stream and infer the relative mass in the progenitor vs. the tidal tails).

- Currently, there is a mild disagreement between the mass inferred from the dynamical model (< 2e6 at 95% confidence) and the mass inferred by the comparison to Pal 5 (> 2e6). Although this level of disagreement does not rule out the possibility of a stream that is consistent with both predictions, I think the above suggestions could help to alleviate some of that discrepancy. It's possible that the Pal 5-based surface brightness estimate is over estimating the required mass of the GC stream. In that case, it would be helpful to know how changes in that preferred mass propagate to the other conclusions of the paper.

- Relatedly, is the surface brightness consistent with a dwarf galaxy stream? It is not as convincing to say that there are possible GC masses that could produce a detectable stream. Instead, it is important to show that there is not a realistic DG progenitor that could produce the observed morphology. I suggest testing this either with simulated streams, or with a MW stream (e.g. Orphan-Chenab). (I expect that this will be difficult to conclude, and that this statement will need to be made in combination with the dynamical model. However I do still think it is worth repeating the surface brightness analysis for a DG progenitor.)

- Are there any statements that could be made about the expected mass function of satellites around a host galaxy of this mass (in comparison to the expected GC population) that could inform the conclusion that this is not a DG stream?

- If you are selecting a progenitor mass that is not right at the peak of your posterior distribution, it would be helpful to show a comparison between the morphology of the stream generated from the selected parameters and from the best-fit values. Would the full range of streams preferred by the dynamical model be observable? I suggest repeating the surface brightness calculation for a sample of stream parameters selected from the posterior distribution.

- In addition to the above suggestions, it would be useful to have more discussion on why the model is inconsistent with a more massive progenitor. Are there assumptions that could be loosened that would allow for a larger mass range? Given the limited observational measurements and the uncertainty in stream models, I think it's important to include more robust discussion of why it really is not plausible that a DG stream could produce the observed morphology. It would strengthen the conclusion of the paper to more clearly describe what would need to change and by how much in order to produce a consistent DG stream model.

Clarification & Minor Comments:
- It is not clear to me how the prominence of the feature is calculated. How exactly are the flux values for the stream and background determined?

- In the caption of Figure 2, I would add a little bit more detail about the error bars. The origin of those error bars was unclear the first time the figure was referenced, and only clarified later in the text.

- Relatedly, what is the approximate scale of the measurement uncertainties of the colors and magnitudes of each point in Figure 2?

- I suggest including the pronunciation of the stream name. This has not typically been included in stream discovery publications, which in many cases has led to confusion among the community. I think it's great to name these systems after terms from diverse cultures around the world, but in order to properly respect the cultures and languages the names are borrowed from, it is important that we pronounce the names correctly.

- In Figure 3, it would help to add the red dots that are shown in Extended Data Fig 3 to the corner plot, since those values correspond to the stream shown in the right-hand panel of that figure.

- Relatedly, in Extended Data Fig 3, it would be nice to have red lines showing the positions of the selected parameter values in the 1D marginalized histograms.

- There is a typo on line 308: "which where included in the fit"

- There is a typo in Code Availability: “streamsculpture”

Referee #2

(Remarks to the Author)
Summary of the key results.
The authors report the discovery of the first extragalactic stellar stream consistent with a tidally stripped globular cluster, identified in deep Hubble Space Telescope imaging of the ultra-diffuse galaxy UGC9050-Dw1. They present HST and CFHT imaging of the galaxy showing a bow-shaped feature next to a bright point source with a signal prominence of 15. The galaxy was first identified and reported in Fielder et al. 2o23 who claimed that most of the GCs likely formed simultaneously during a past dwarf merger event. The authors here suggest several observational lines of evidence supporting the globular-cluster-origin interpretation, including the stream’s narrow morphology, coherent structure, colour, surface brightness, and its apparent association with a compact source interpreted as the progenitor cluster.
Using generative modelling of the stream, the authors provide the first stream-based constraint on the dark matter halo of an ultra-diffuse galaxy. Their modelling places limits on the inner density profile of UGC9050-Dw1 and yields dynamical bounds consistent with independent halo mass estimates derived from globular cluster counts. The inferred properties of the stream are also consistent with expectations for a globular cluster progenitor within such a galaxy.
The authors argue that extending stream analyses beyond the Milky Way opens a new avenue for probing dark matter in low-mass, low-surface-brightness galaxies. They emphasize that previously suggested extragalactic globular-cluster–origin streams have either been ruled out or lacked statistically significant detections, making this the first robust example. The work therefore establishes a new method for testing competing models of dark matter by leveraging stellar streams in external galaxies.
The central claim of the paper rests on the identification and interpretation of a very faint structure as a stellar stream associated with point sources in a low surface brightness galaxy at a distance of approximately 35 Mpc, which the authors suggest may be globular clusters belonging to the system. While the authors demonstrate that the faint structure is present in both the HST and CFHT imaging and is therefore unlikely to be a noise artifact, the evidence supporting its interpretation as a stellar stream remains weak. The color–magnitude diagram analysis does not provide convincing support for a globular cluster origin of the point sources, and much of the evidence presented is circumstantial. Alternative explanations, including projection effects or dust-induced features that can mimic similar colors and morphologies, are not sufficiently ruled out. Although future JWST imaging may offer valuable constraints on the nature of this structure, more rigorous tests using the existing data are required before the paper can be recommended for Nature. Not enough information is provided on the methodology of the stream detection to judge the validity of the result.
Originality and significance: if not novel, please include references.
The detection and use of stellar streams as probes of dark matter has been extensively developed in the context of the Milky Way, where thin, low–surface-brightness globular-cluster streams have been shown to provide sensitive constraints on the halo potential and small-scale structure. However, identifying such streams in external galaxies has long been considered beyond current capabilities, given their extreme faintness and narrow morphology. Even within the Local Group, previous extragalactic globular-cluster–origin stream candidates have not held up to scrutiny or lacked statistically significant detections above the background. The prevailing view had been that facilities such as Rubin Observatory would be required before such structures could be reliably observed beyond the Milky Way.
In this context, the identification of a compelling extragalactic globular-cluster stellar stream candidate in deep HST imaging represents a significant and novel advance. If confirmed, such streams offer a powerful and currently untapped probe of globular cluster ages and metallicities (thought to trace early stages of galaxy assembly), the accretion history of the host galaxy, and the inner density profile of low-mass dark matter halos. These are topics of central importance to galaxy formation and to distinguishing between competing dark matter models. The ability to extract this level of information from an ultra-diffuse galaxy, using stream-based dynamical modelling, would mark a major step forward and could open a new regime for studying extragalactic halos and age–metallicity relations.
Data & methodology: validity of approach, quality of data, quality of presentation.

Detection of the stellar stream candidate
Lines 263–266: In the CFHT data, the putative stream is detected only in the g band and not in the u, r, i, or z bands. While the integration time and seeing are reported for the g-band image, corresponding information for the other filters is not provided. It is therefore unclear whether the g band is the deepest image in the survey or simply has the most favorable image quality.
The authors should clarify whether the g band represents the deepest or highest-resolution CFHT data. Quantitative upper limits on the surface brightness or flux of the feature in the u, r, i, and z bands should be provided. These limits should then be compared to expectations for a stellar stream originating from a globular cluster, based on Milky Way streams, and to the surface brightness measurements derived from the HST data. In addition, it would be helpful to know whether the authors have attempted to identify the feature in stacked or combined u, r, i, and z images.
To allow an independent assessment, image cutouts centered on the candidate stream region should be shown for all five CFHT filters, presented with consistent scaling.
Prominence of the feature
The manuscript refers to the prominence of the feature, but it is not clear in which image or filter this quantity is calculated. The authors should specify the exact image used and report the prominence consistently across all available CFHT and HST filters.
Lines 313–314: The mask shown in Extended Data Figure 2 is used for both prominence and color measurements, yet the procedure for constructing this mask is not described in sufficient detail. In particular, the authors should clarify which image is used to define the manually selected points along the track and how sensitive the results are to this choice.

Properties of the stellar stream candidate
Lines 135–142 and 340–353: The stellar stream is characterized as having a mass approximately 20 times that of Palomar 5, based on stellar population simulations anchored to Pal 5. It is unclear whether alternative stellar populations have been explored. For example, a disrupting young star cluster could plausibly produce a brighter feature with a lower total mass. The authors should test a broader range of stellar population models and discuss how strongly their conclusions depend on the Pal 5 analogy.
The possibility that the feature is associated with dust is not adequately addressed. The morphology and colors could also be consistent with a dusty region, particularly given the apparent void immediately below the feature, which could correspond to a region of high attenuation. This is especially relevant if the host system is a dusty, young dwarf galaxy. The authors should test whether dust attenuation could plausibly reproduce the observed morphology and colors.
Color and CMD analysis
Figure 2: It is unclear what conclusion the color analysis of the stream candidate is intended to support. The comparison is limited to the progenitor candidate within the same galaxy, which does not provide sufficient external context. For comparison, the authors should include color–magnitude diagrams of well-studied stellar streams in the nearby universe. For example, Bonaca et al. (2012) present CMDs of Milky Way globular cluster streams with comparable widths.
Similarly, the globular cluster candidate selection box from Fielder et al. (2023) should be complemented with comparison samples of Milky Way globular clusters, young star clusters in the Milky Way, and clusters in nearby galaxies. Without these comparisons, it is difficult to assess how diagnostic the presented CMD is.
More broadly, the analysis relies on a single color–magnitude comparison. Given the availability of multi-band data, the authors should demonstrate whether additional colors show consistent localization. Recent work, such as arXiv:2306.06164, illustrates the power of multi-color constraints in establishing stellar population coherence. The authors should also quantify how much age and metallicity variation is permitted within the observed color scatter, and whether the test shown is sufficient to support the conclusion of a common stellar population.
If reddening is present, this further complicates the interpretation. The authors should explicitly address the possibility of internal or foreground reddening and discuss how it affects their stellar population inferences.
Association with the host galaxy and signal-to-noise
Several key assumptions remain insufficiently justified. The stream identification appears to rely largely on visual inspection, and it is unclear how objective the shape determination is. The quoted signal-to-noise ratio of 15 requires further justification. Given the lack of a priori evidence that all segments of the feature belong to the same stellar population, the signal-to-noise should be evaluated region by region along the stream.
It is also not clear how the globular cluster candidates and the stream are confirmed to be physically associated with the ultra-diffuse galaxy rather than being chance projections. The manuscript should more rigorously address this point.
Modeling and statistical robustness
For Figure 3, the authors present a single realization of the modeled stream. It would be more informative to show multiple samples from the posterior distribution to illustrate where the spatial constraints are tight and where they are uncertain. The observational data should also be marked more clearly on these figures.
The modeling does not appear to account for surface brightness fluctuations or clumpiness along the stream. The authors should discuss whether such effects could bias the inferred morphology or width.
Statements such as “we find that a GC stream origin of the observed feature is dynamically plausible” would benefit from a quantitative assessment of confidence. Is it possible to place statistical bounds on this plausibility, rather than relying on qualitative agreement?
Similarly, the claim that the perpendicular brightness profile resembles a stream more than a shell would be strengthened by explicitly showing both profiles in one-dimensional plots for direct comparison.
Presentation
Finally, Figure 1 does not clearly highlight the feature of interest. For comparison, Figure 1 of Fielder et al. (2023) provides a much clearer visualization of the relevant structure. Improving the presentation of the discovery image would significantly aid the reader.
Appropriate use of statistics and treatment of uncertainties.
The color–magnitude diagram on Fig 2 lack y-error bars. Do Fielder+23 report error for their CMD?
Several key results appear to rely on visually selected regions or manually defined masks, yet the sensitivity of the results to these choices is not explored.

Conclusions: robustness, validity, reliability.
Please see the above sections.
Suggested improvements: experiments, data for possible revision
If possible, the authors should analyze the candidate stream in spatially distinct segments rather than treating it as a single structure. The signal-to-noise ratio should be evaluated independently along different portions of the feature to assess whether the detection is robust throughout. Photometry from these segments should then be added to the CMD analysis to test whether they are all consistent with a common stellar population within uncertainties. This would provide a more statistically grounded assessment of whether the feature represents a coherent stellar stream.

Referee #3

(Remarks to the Author)
I co-reviewed this manuscript with one of the reviewers who provided the listed reports.

Referee #4

(Remarks to the Author)
I read with interest the paper "Evidence for the First Globular Cluster Stellar Stream beyond the Milky Way" by Kiel Holm and collaborators. The manuscript presents the detection of a possible globular cluster stream in an ultra-diffuse dwarf galaxy UDG9050-Dw1 and uses these data to study the dark matter properties of the host.

The article is interesting and well written, but I have three main issues with it.

First, the authors claim that theirs is the first detection of a globular cluster stream beyond the Milky Way. However, quite recently Poulain et al. 2025 (Nature 640, 902) showed clear evidence of stellar streams associated with merging nuclear star clusters in a sample of dwarf galaxies studied with HST imaging. Furthermore, in the nearby Andromeda galaxy, stellar streams have been known to be associated with star clusters for many years. Indeed, Pearson et al 2022 (ApJ 926, 166; led by a co-author of this paper, and whose secondary title in the arXiv version of that paper is "GC streams in M31") present a gallery of GC stream candidates. So I am somewhat baffled by the main claim that underlies the present manuscript.

Second, the paper uses a method to analyse the properties of the host galaxy which has not yet been refereed. This makes it very difficult to judge the validity of the claims without a thorough examination of that software. My intuition from long experience of modelling stellar streams is that the algorithm cannot possibly work as well as is claimed, since the observed structure is (at best) extremely noisy, no kinematic information is available, and it is only seen in projection. I strongly suspect that the authors are being misled by a perfect match of their model and artifical test data.

Third, I find the images presented here far from convincing, and far from the claimed 6-sigma detection level. In ED Fig 1 one can see how noisy the image is, and a comparison to Fig 1 highlights that one can identify several linear features by eye over a large field, but which are probably suprious noise alignments. The structure presented in this article looks (to me) possibly real, but I suspect the 6-sigma claim almost certainly ignores the look elsewhere effect.

My conclusion is that the paper is interesting, and presents a glimpse into exciting future science, but the authors' case is not watertight, and the community would benefit from the claims being substantially toned down.

Version 2:

Reviewer comments:

Referee #1

(Remarks to the Author)
The authors have thoroughly responded to all comments, strengthening the conclusion that the stellar stream is of globular cluster origin. I therefore recommend this paper for publication in Nature without further revision.

Referee #2

(Remarks to the Author)
I have read the rebuttal provided by the authors for all three referees and the revised manuscript. I am happy with the additional quantitative analysis provided, as well as the new figures. While I agree with referee#3 that the claim should be toned down (I noticed that the claim for "6-sigma detection" has been removed), this is an interesting finding that is worth publication. Hence, I recommend the article for publication.

Referee #4

(Remarks to the Author)
The authors have addressed my concerns, and so I am happy with the new version of the manuscript.

Open Access This Peer Review File is licensed under a Creative Commons Attribution 4.0 International License, which permits use, sharing, adaptation, distribution and reproduction in any medium or format, as long as you give appropriate credit to the original author(s) and the source, provide a link to the Creative Commons license, and indicate if changes were made.
In cases where reviewers are anonymous, credit should be given to 'Anonymous Referee' and the source.
The images or other third party material in this Peer Review File are included in the article's Creative Commons license, unless indicated otherwise in a credit line to the material. If material is not included in the article's Creative Commons license and your intended use is not permitted by statutory regulation or exceeds the permitted use, you will need to obtain permission directly from the copyright holder.
To view a copy of this license, visit https://creativecommons.org/licenses/by/4.0/

Referee #1:

Summary & Significance: This paper presents the discovery of globular cluster stream around a dwarf galaxy located 35 Mpc from the Milky Way, as well as a dynamical constraint on the halo mass of the host ultra-diffuse galaxy. Both of these results have significant implications for the fields of galaxy evolution and the astrophysical study of dark matter, and therefore are of clear interest to a broad scientific community. I have some suggestions for additional analysis and discussion, described below. After these comments are addressed, I recommend the publication of this paper in Nature.

Interpretation & Conclusions: My primary comments are related to the question of whether the stream is the remnant of a disrupted globular cluster or dwarf galaxy. The discovery of a globular cluster stream would be novel and deserving of publication in Nature, while the discovery of a dwarf galaxy stream (of which many are known) would not. Overall, I find the analysis convincing, but I have requests for clarification and suggestions for additional tests to further strengthen the conclusion that this is in fact a globular cluster stellar stream. Generally, I think it is important to show that the stream is not consistent with a dwarf galaxy origin, in addition to showing that it is consistent with a globular cluster origin.

We thank the referee for the feedback and suggestions, which we have considered and answered in detail below.

Analysis & Statistics:
Q1) The measurement of the width of the stream is a key piece of evidence for its globular cluster origin. Since the surface brightness of the stream decreases at larger distances from the central track, it would be good to perform additional tests to ensure that detection limits are not leading to an artificial decrease in the measured width. I suggest injecting simulated streams of different widths into the data and testing your ability to recover the correct width. As mentioned above, I think it is important to demonstrate that the data is not also consistent with a larger width.

1A1)
We agree that such tests are valuable. Based on the suggestions in Q5 on investigating a wider range of isochrones than Pal 5's and the suggestions in Q6 to mock observe the model streams, we have decided to focus on three different model streams in this report, one of which mimics a dwarf stellar stream.

1. **Lower bound stream** (see middle panel of Fig 3 in the revised manuscript)
    - mprog = 1.65 x10^5 Msun.

        - This new stream model selected within the 1sigma bounds of our posteriors from X-stream but with a lower mass progenitor, corresponding to the lower bound progenitor mass which can still produce an observable stream based on our new surface brightness calculations (see answer to Q6).
    - Age: 8 Gyr
    - [Fe/H] =-1
    - Number of stars: 280,000 stars
        - Also motivated from the surface brightness calculations, which generates a population of 281.000 stars (with total mass of 1.24 x10^5 Msun in the stream arms) for our brightest isochrone. See our answer to Q5 for a discussion of the mass ratios between the stream and progenitor in our analysis.

2. **20 x Pal 5-like stream** (right panel of Fig 3):
    - mprog = 2 x 10^6 Msun
        - This is the same stream as in the submitted manuscript which was shown to be observable in the HST data with Pal 5's mass loss history and isochrone
    - Age: 11.3 Gyr
    - [Fe/H] = -1.3
    - Number of stars: 1.170.000
        - motivated from the surface brightness calculations, where a simulated population of 1.17x10^6 stars reaches the sufficient brightness.

3. **Dwarf-like stream** (see new extended data fig 6)**:**
    - mprog = 2 x 10^7 Msun (similar to SGR stellar mass)
    - Age: 11.3 Gyr
    - [Fe/H] = -1.3
    - Number of stars: 1.170.000
        - Same number of stars as above to mimic a dwarf progenitor where the extra mass is in dark matter. We use an identical stellar population as for the 20 x Pal 5-like stream (for easy comparison).

For each stream model above, we sample an IMF up to the stellar mass that survives at present day, and assign magnitudes in the F555W and F814W Hubble bands calculated from PARSEC1.2 isochrone of age and metallicity selected from the scan described in A6.

For each star in this artificial stellar population we convolve the magnitude with a EPSF described by Mitchell Revalski (2025) (https://spacetelescope.github.io/hst_notebooks/notebooks/WFC3/point_spread_function/hst_point_spread_function.html) which uses PSF models from Jay Anderson (https://www.stsci.edu/~jayander/HST1PASS/LIB/PSFs/STDPSFs/). The spread flux from all the stars in the model stream is added up, poisson noise is added, and then the mock is injected into the two HST images. We inject them in the same approximately empty area near the Oyashio stream, since it allows for easy visual comparison.

Below we show the result of our analysis for each of the three streams. We have also added this to the appendix of the paper.

The mock observations of the model streams for the lower bound stream (left) and the 20xPal 5-like stream (middle) are faintly visible and look similar to the observed Oyashio feature in the data. Note that the more diffuse stars at the end of the model streams in are not clearly visible for the mock streams.

The mock observations of the model dwarf-like stream (with mass 10^7 M_sun) is too faint for our width measurement to converge, which makes sense, since it is the same number of stars but spread out across a larger area, yielding a lower surface brightness. We thus conclude that a dwarf stream with the same stellar mass as the upper bounds of our dynamical models, would not produce an observable stream.

If we increase the brightness, or equivalently, the number of stars, in the dwarf-like stream until the surface brightness is comparable to the observed data, the width of the stream is noticeably wider than the observed Oyashio stream (see right panel of extended data fig 6, where we have scaled up the brightness by a factor of 5).

We conclude that a dwarf stream would either be too faint to be observed with a viable stellar mass, or be observed as wider than our detected Oyashio feature. We have updated the manuscript to discuss this and the caveats of this analysis (particle spray streams, mass loss history assumptions, and stellar population assumptions).

This additional plot is included below. It is clear to the eye as well, that the dwarf-like stream would appear wider than the observed one.

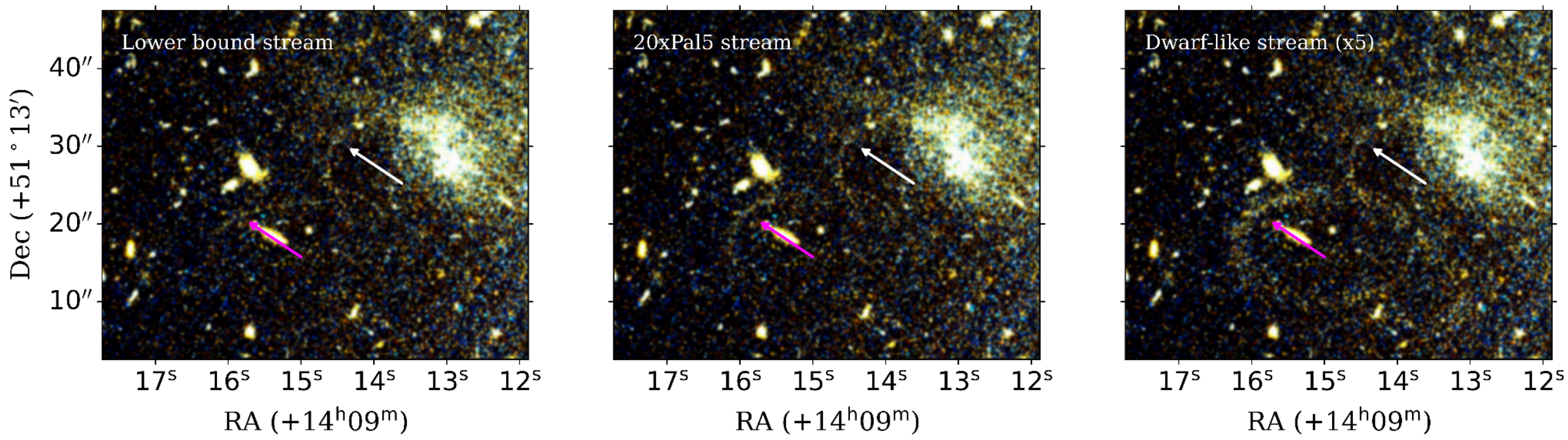

The three mock observations. *Left: The lower-bound stream, Middle: 20xPal5 stream, Right: Dwarf-like stream. The white arrows point to the actual stream candidate, and the magenta arrows to the injected mocks. These figures are also presented in the manuscript as Extended Data Figure 6.*

Q2) In addition, it could be helpful to include additional information about the confidence of the measured properties of the globular cluster progenitor. For example, how does the inferred size and luminosity depend on the selection region?

1A2)

We thank the referee for the suggestion. The expected extent of the cluster is similar to the pixel scale without PSF effects, so we do not expect to be able to measured the size independently. In terms of the mask used, the low number of pixels hinder a detailed profile analysis like fitting a 2d Gaussian. Instead, we have varied the width of the circular mask of the cluster, to explore its convergence. At some width w, the additional flux achieved by increasing to w+dw is comparable to the addition in background, and the corrected flux evens out (see e.g. Johnson et. al, 2012 (doi:10.1088/0004-637X/752/2/95) for an example of similar methods).

In the figure below we have illustrated this effect. The flux inside the mask is normalised to the mean, for easy comparison between the two bands. We see that after a width of around 30 pixels (256 pc), extra area does not lead to additional flux, and inside 20 px the gradient starts to decrease.

We stress that this width is far too large to correspond to the half-light radius of a few pc that is commonly assumed for GCs, but that is not what it measures. If we were to crudely measure the half-light ratio here, not accounting for any PSF or other observational effects, it would be enclosed in 12-13 pixels (~100-110 pc). We want to direct the referees' attention to the other GC candidates in the image, one of which is highlighted in Extended Data Figure 3, which have comparable, if not larger, apparent sizes, although they are assumed to be point sources at this resolution scale (Fielder et al. 2023). Thus, this is not a suitable way of measuring the actual size of the cluster, but

it can provide basis for a discussion of the choice of width in the mask used to measure the cluster's brightness.

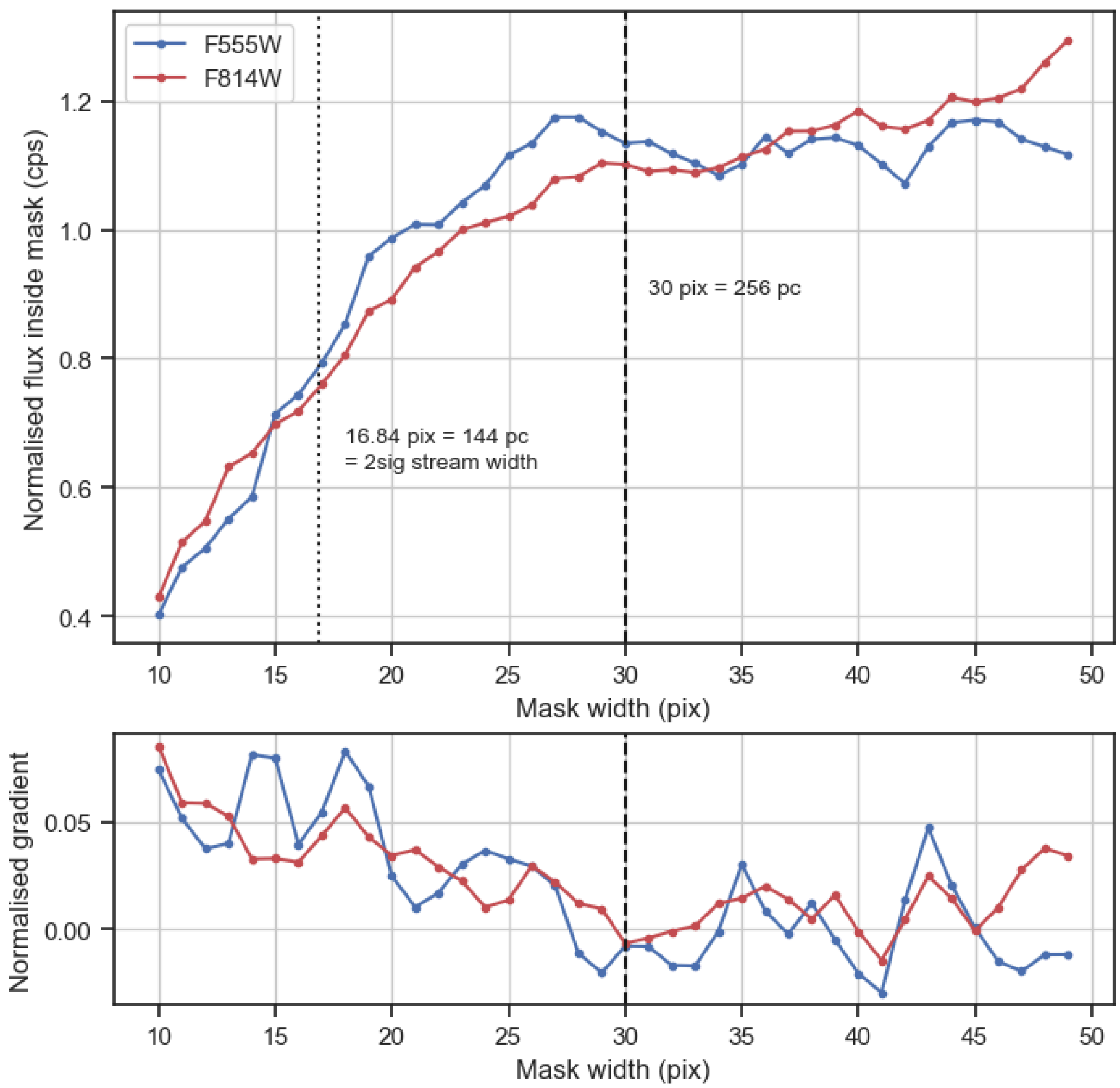

Q3) The significance of the similarity in the colors between the stream and the GC candidates would be easier to interpret with some qualitative discussion of the probability of the colors of the candidate stream and GC matching in the case that they are not associated.

1A3)

We agree with the referee, and have added an illustration of the distribution of MW GCs in the CMD, showing another possible spread of similar objects. A single color measurement for a GC allows for a range of stellar populations, and the CMD presented here is as such not intended to tightly constrain the properties of the population. The intent is rather to show that both objects fall within the CMD selection criteria of Fielder et al., and that their colors are consistent with each other.

We have also included a brief comparison with the colours of MW streams and clusters in the text:
This is slightly bluer than the average GC in the MW \citep{Harris1996, Larsen2001}, which aligns with the findings in \citet{Fielder2023}.

Q4) This sentence is not clear to me: “Pal 5 contains 3000 stars in the DESI g band between magnitudes 23 and 20, which we use as a threshold for our stellar sample.” Do you mean that you use that sample of stars, combined with an IMF, to determine the total luminosity of the stream beyond these magnitude limits?

1A4)

Yes. We sample randomly from the IMF until we reach 3000 stars between 23 and 20 in the g band, and then use the entire sample (inside and outside that range), truncated in the high mass end to the limit of the isochrone (i.e., the mass of the most massive surviving star with this age) to determine the luminosity of the stream.
Since we have expanded out isochrone methods based on the suggestions in Q6 we have updated that entire section of Methods. See response A6 for manuscript changes.

Q5) How does the level of disruption (i.e. the length of the stream) affect the estimated surface brightness? A stream that is less massive and less disrupted could have the same surface brightness as a stream that is more massive and more disrupted. The decision to assume that the disruption level is similar to Pal 5 should be more clearly motivated, or a broader range of stream lengths should be accounted for in the calculation.

1A5)

The mass loss of the stream is not possible to determine from the available data. However, we agree that assuming it is like Pal-5 may not be the optimal approximation. In addition to our analysis from Pal 5, we now also use the brightness ratio between the cluster candidate and stream candidate as a proxy, and compare the total brightness of the stream in F814W and F555W to that of the stream+cluster. This gives a ratio

M_stream/(M_stream+M_cluster) ~ 0.60

(0.62 in F555W and 0.58 in F814W).
If we take into account that we only observe one arm, and assume that the arms are symmetrical, the mass ratio becomes

(2*M_stream)/(2*M_stream+M_cluster) ~ 0.75

(0.76 and 0.74, respectively). We use this ratio as our measurement of the level of disruption throughout the analysis. While there are several assumptions tied to this, it is the most data-based estimate we can make.

We also refer the referee to our analysis in A1. The density of stars decreases along the length of the stream, so deciding when it “ends” is, especially with faint, integrated light, a somewhat arbitrary choice. In our answer to the next referee’s Q11 we show an analysis of the width and signal prominence performed in segments along the stream, and conclude that no one segment dominates the values. This indicates that our choice to base the length on the by-eye visibility of the feature does not affect our results to a significant degree.

In modelling the stream, the length is a free parameter and that X-stream does not penalize fits with longer streams than the input data. Therefore, the length of our control point area serves as a lower bound for the length.

In the manuscript we have added:
Since we do not know the detailed mass loss history of the GC candidate, in order to get the total mass of the stream and progenitor, we assume that the brightness ratio between the cluster and stream is an appropriate proxy for the mass ratio. Assuming that the two stream arms are identical, this gives a ratio of M$_{\rm stream}/$M$_{\rm total} \sim 0.75$, which we use to scale the inferred stream mass to a total progenitor mass.

Q6) When sampling from an isochrone to generate a stellar population, are the age and metallicity selected to match Pal 5? The paper mentions that the candidate stream is consistent with being younger and more metal rich (and therefore brighter) than Pal 5. Why not sample from a broader range of isochrones to test the detectability of streams across the range of stellar population parameters that are consistent with the measured color?

1A6)
We thank the referee for the feedback regarding the choice to compare to Palomar 5 stream. We agree that it would be better to compare to a suite of stellar streams, with different mass loss rates and isochrones.

The most general way to address this is to simply measure which stellar mass it would take for the stream to have the measured surface brightness in the available bands. It still requires choices about the IMF, stellar evolution, and mass loss history, and ignores

effects of possible mass segregation in the progenitor cluster. We have used the IMF measured for the Pal-5 cluster remnant, which is a shallow powerlaw (Grillmair 2001), and isochrones calculated using the PARSEC 1.2 model. The IMF mass range is truncated to match the isochrones, which, since significant tidal disruption requires older populations, go from around 0.09 to 1.2 Msol. Anything more massive than that is assumed to have died.

With this, we sample a stellar population and calculate its total luminosity in the relevant observational bands for 30 different isochrones, varying the age from 8 to 12 Gyr, and the metallicity [M/H] from -3 to -0.5. Fielder+ 2023 finds the limits age > 1.5 Gyr and [M/H] < -0.57 for the GC population in UGC9050-Dw1. This age is too low to allow for significant amounts of tidal stripping, so we set the lower bound to a more realistic 8 Gyr.

We vary the total mass of the population sampled, and use an area of 16 arcsec^2 (twice the area of the stream mask used on the data, since we only see one arm) to calculate the surface brightness. We compare to the surface brightness measured in the HST data, which is 27.00 mag/arcsec^2 in F555W and 26.08 in F814W (across the 8 arcsec^2 mask).

The lowest total mass to give surface brightnesses \geq that of the data is 6.2e4 Msol, which is bright enough in the youngest isochrones. We show here an illustration for 7.0e4 Msol, highlighting (with black text) in H814W that several isochrones yield a surface brightness of 26.1 mag/arsec^2 or brighter. All these also yield a F555W surface brightness in agreement with data.

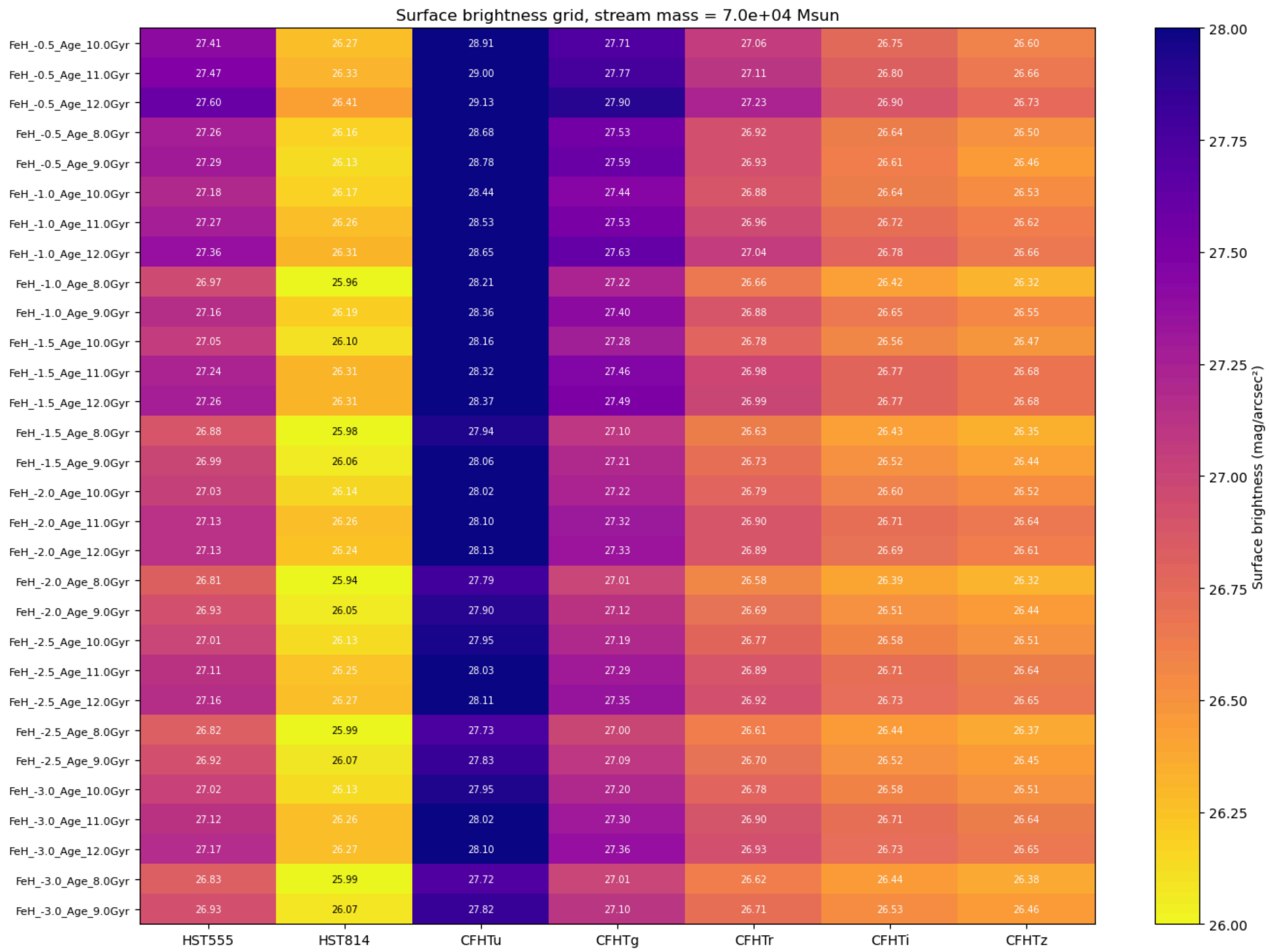

The result of our isochrone scan*. The isochrones on the vertical axis and their brightness in each telescope filter on the horizontal. The color and numbers in each cell is surface brightness over an 16 arcsec^2 area, and black numbers indicate brighter than the observed F814W surface brightness.*

To make that measure meaningful, we need to compare it to values from the literature. For example, Palormar 5, has recent estimates of the total current mass between 1.2 and 9.0e4 Msol (Ibata2017 and Price-Whelan2019), and with a GC remnant mass estimate of only 4300 Msol (Ibata2017) this leaves the stream mass estimate to range from 7.7e3 to 8.6e4 Msol. Our mass estimate for these populations fall within those boundaries. The isochrone needed to get this mass has a different age and metallicity than Palomar 5, but this example serves to show that a stream with a Palomar-5 like mass could produce this feature if the stellar population is a few Gyrs younger.

To relate it to some total progenitor mass, we would need details on mass-loss history or mass ratio between the stream and GC, which are unavailable. By using the assumptions described in A5, we can scale the lower bound stream mass of 6.2e4 M_sun to a total mass of the progenitor system. If we take into account that this is only

one arm, and assume the arms are symmetrical, we use the mass ratio of ~0.75 and the total mass becomes 1.65e5 Msol.

We use this population as our lower bound to what is possible: An isochrone of age 8 Gyr, [M/H]= -1, and total initial progenitor stellar mass 1.65e5 M_sol.

This approach gets rid of the reliance on the Pal5 star count and isochrone choice. It assumes a stellar IMF that is identical in the stream and the GC, and that it is similar to the measured IMF of Pal5. Different IMF descriptions will give varying results, but we find this to be the least arbitrary choice.

We caution that the comparison between the two models we call the lower bound-stream and 20xPal5-like stream is not a direct apples-to-apples comparison. The “lower bound” is sampled until a population of a certain stellar mass is reached, which is bright enough. This total mass is used directly as the stellar mass in the stream arms. The “20xPal5” stream is sampled until a population with a certain *starcount* is reached, which makes it bright enough. The inferred total progenitor mass is found by scaling a separate estimate of Pal5’s initial mass to this starcount, which gives that 20x M_pal5 = 2e6M_sun. But the total mass of the stars in the simulated population is significantly smaller than this number, even if scaled by large mass ratios. The total mass of the simulated population does agree within the range of estimated pal5 stream mass of 7.7e3 to 8.6e4 Msol from Ibata2017 and Price-Whelan2019 mentioned above.
These two simulated populations represent two different approaches to estimating the mass, and can be taken as examples of the extremes: The “lower bound” population assumes that every star is still within the measured region, and the “20xPal5” assumes the same mass-loss history as Pal5 and describes the initial mass of the cluster before any disruption occurs.

We have updated this paragraph in Methods and amended the main text to reflect this new approach.

Q7) In addition to using Pal 5 as a comparison to calculate surface brightness, it would be interesting to use the stream from the dynamical model. I suggest first doing a rough surface brightness calculation along the stream, to determine whether the stream would be detectable given the expected mass and level of disruption, and second whether a larger extent of the stream is expected to be observable (does the surface brightness of the tail drop off quickly enough? If not, is there a proposed explanation?). You could also consider injecting the simulated stream into the actual image to similarly test whether the observed morphology would match the data. Of course, all of this should be

presented with clear caveats, and I would not expect the simulated stream to match perfectly, but I think it would help strengthen the interpretation.

1A7)

We refer the referee to the answer to Q1, our descriptive text in the Data Analysis section and for the new Extended Data Figures where we illustrate the differences in three mock observed streams and discuss the caveats. Specifically, we also point the referee to the middle panel of the new Extended Data Fig 6, where we show the 20xPal 5-like stream injected to the data. While the simulated stream is longer than the actual data, once the stream is injected and mock observed in the data, the end of the tail is no longer visible, as it is too diffuse.

Interestingly, the new lower bound stream in the middle panel of Fig 3, from a lower mass progenitor produces an observable stream which does not exceed the extent of the observed data by much. Note also that X-stream does not penalize fits with longer streams than the input data, and the ends of stellar streams are more diffuse and often made up of lower mass stars (Balbinot et al. 2018), which could help explain why we don't see the extension in the data even if the initial progenitor mass was higher than 10^5 Msun.

Q8) By more robustly estimating the detectability of the simulated streams, you may be able to make some additional statements about the properties of the GC progenitor (e.g. if you are able to estimate the length of the stream and infer the relative mass in the progenitor vs. the tidal tails).

1A8)

We thank the referee for this suggestion. As mentioned in A5 the relative mass is difficult to obtain, but if we use the relative brightness in the HST bands as a proxy for mass, the stream is ~1.5 more massive than the current GC.

We believe that getting a reliable length measurement is unrealistic with the available data, since a segmented test of prominence is heavily dependent on the length of the segments. In 2A11 we outline a significance test performed in segments along the stream to show that no single segment is dominating. If we extend this much further the fit on the furthest segments converge unreliably or not at all, indicating that the visual end of the stream is a good estimate. As mentioned above, a stream gets more diffuse further from the progenitor, so the visible length of the stream is a lower bound.

When modelling the stream with XStream we choose a slightly conservative length, since, as mentioned in A5, it serves as a lower bound also in the modeling.

Q9) Currently, there is a mild disagreement between the mass inferred from the dynamical model (< 2e6 at 95% confidence) and the mass inferred by the comparison to Pal 5 (> 2e6). Although this level of disagreement does not rule out the possibility of a stream that is consistent with both predictions, I think the above suggestions could help to alleviate some of that discrepancy. It's possible that the Pal 5-based surface brightness estimate is over estimating the required mass of the GC stream. In that case, it would be helpful to know how changes in that preferred mass propagate to the other conclusions of the paper.

1A9)

We appreciate this discrepancy being pointed out. With the deeper analysis presented in A6 we show that a lower progenitor mass is possible with a younger stellar population, which also more consistent with the findings of the bluer, younger populations of GCs reported by Fielder et al. 2023.
We have loosened our description of the mass estimate to not be as tightly tied to the Pal 5 analogy. Now we instead set a lower limit for the total mprog of 1.65 x 10^5 Msun. This is well within the bounds from the dynamical model. We have changed the text to reflect this.

Q10) Relatedly, is the surface brightness consistent with a dwarf galaxy stream? It is not as convincing to say that there are possible GC masses that could produce a detectable stream. Instead, it is important to show that there is not a realistic DG progenitor that could produce the observed morphology. I suggest testing this either with simulated streams, or with a MW stream (e.g. Orphan-Chenab). (I expect that this will be difficult to conclude, and that this statement will need to be made in combination with the dynamical model. However I do still think it is worth repeating the surface brightness analysis for a DG progenitor.)

1A10)

We refer the referee to our answer to Q1 where we compare mock observations of simulated GC and DG streams, and compare their detectable width.

The surface brightness of MW streams from GC and DG progenitors are not necessarily distinctively different (see e.g. Shipp et al. 2018, where the Indus and Chenab DG streams have comparable SB to the GC streams). The main difference is in the width,

which is larger for a DG stream, meaning the required stellar mass to produce a certain SB is also larger (since the area increases).
Since DGs often include several stellar populations, performing a similar calculation for a DG progenitor would only add degrees of freedom by combining different isochrones.

While the surface brightness analysis alone does not put strict upper limits to the mass of the system, a common argument against the possibility of detecting extragalactic GC streams is that one such would have too low a surface brightness. With this exploration of isochrones we argue that it is possible for a GC with certain populations to produce a brightness equivalent to what is observed here.

In the X-Stream simulations we do not restrict the prior to only allow for a GC progenitor, but test a wide range of dynamical masses. The constraints exclude total progenitor masses (including any dark matter) above 2.6*10^6 M_sun with 95% confidence, which is below the most commonly accepted span of dynamical DG masses.

Q11) Are there any statements that could be made about the expected mass function of satellites around a host galaxy of this mass (in comparison to the expected GC population) that could inform the conclusion that this is not a DG stream?

1A11)
While there are studies showing GC and halo mass relations for UDGs (e.g. Harris et al. 2017), we are unaware of similar studies for satellite counts. Dooley et al. 2017 explored the expected number of dwarf satellites for isolated LMC hosts, comparable to the estimated halo mass of UDG 9050-Dw1, finding 2-12 satellites above 10^4 Msun (their Fig. 1), and 2-7 above 10^5 Msun. This is based on abundance matching, isolated hosts, and not specific to UDGs. We are unaware of any studies claiming to have found dwarf satellites around UDGs, but would be happy to investigate this further if the referee has specific suggestions.

Q12) If you are selecting a progenitor mass that is not right at the peak of your posterior distribution, it would be helpful to show a comparison between the morphology of the stream generated from the selected parameters and from the best-fit values. Would the full range of streams preferred by the dynamical model be observable? I suggest repeating the surface brightness calculation for a sample of stream parameters selected from the posterior distribution.

1A12)

This is a great point. As per the SB calculations presented above in A6, using different ages and metallicites, a progenitor cluster with as low of a mass as 1.65 x 10^5 Msun could be observable. As discussed in our answer to Q1, we have updated Fig 3 to now include two different models: One with this new lower bound (Mprog = 1.65x10^5 Msun) and the one we used before where we assumed 20xPal 5 like mass and age/metallicity. Both of these streams are within the 95% credible regions in the posterior and produce good fits to the data.

Note that the progenitor mass limit is now an upper limit, and not a peaked posterior. This is due to updates to the X-stream code in the referee stage of Nibauer & Pearson 2025 (it has now been accepted for publication). Since they sample a negative KL-divergence, the density of samples can be different from the true posterior and can suppress sharp peaks in the probability of a model stream. To correct for this, posterior constraints are now derived by constructing bins in parameter space and taking the average weight of the samples in each bin. Contours are then drawn using the average weight in each bin. This way, the weights inform the drawing of contours rather than the sampling density of the negative KL-divergence. We have updated all plots in this submitted manuscript to reflect this change. The only difference is that the mprog is now an upper limit rather than a constraint (see Figure 3 and extended data Figure 4), and that some of the quoted 68/95% confidence regions are slightly wider.

We have also added a new appendix figure (extended data figure 5) showing more examples of good fits and examples of inconsistent fits. The main difference in a lower progenitor mass fit is to produce a thinner, shorter stream. The inconsistent fits often only partially overlap with the observed data (incorrect orbits, much wider streams, different curvatures etc.)

Q13) In addition to the above suggestions, it would be useful to have more discussion on why the model is inconsistent with a more massive progenitor. Are there assumptions that could be loosened that would allow for a larger mass range? Given the limited observational measurements and the uncertainty in stream models, I think it's important to include more robust discussion of why it really is not plausible that a DG stream could produce the observed morphology. It would strengthen the conclusion of the paper to more clearly describe what would need to change and by how much in order to produce a consistent DG stream model.

1A13)

We refer the referee to our previous answers and the two new Extended Data Figures 5 and 6 showing examples of bad fits and that a dwarf type stream would be observed to be wider. We have adapted the manuscript to discuss the new extended data figures and why wide streams get disfavored. In summary, X-Stream works by evaluating a smoothed density representation of each model stream at the data control points. Streams wider than the data have excess density inconsistent with the control points, thereby disfavouring wider models. We have added this last sentence to the methods section.

We also refer the referee to Sec. 4.1 of Nibauer & Pearson, where the limits from X-Stream are discussed in detail. Note also that Chemaly et al. 2026 independently finds it possible to constrain halo and progenitor parameters from extragalactic stream imaging and reproduce the general findings of Nibauer & Pearson.

In our paper, we allow for wider stream masks, we mock observe the streams (new extended data figure 6), and all our tests indicate a GC stream not a dwarf stream.

Clarification & Minor Comments:
Q14) It is not clear to me how the prominence of the feature is calculated. How exactly are the flux values for the stream and background determined?

1A14)

Thank you for pointing out this confusion. We have clarified the wording of that section: *To quantify the prominence of the stream candidate, we sum up the counts in the on-stream mask and in the off-stream background masks. We subtract the mean background from the signal, and compare this to the standard deviation of the backgrounds.*

Q15) In the caption of Figure 2, I would add a little bit more detail about the error bars. The origin of those error bars was unclear the first time the figure was referenced, and only clarified later in the text.

1A15)
We have changed the caption:
The errorbars on the colours are the magnitude uncertainties, combined with the standard deviation between the background areas, which reflect the uncertainty tied to choice of background.

Q16) Relatedly, what is the approximate scale of the measurement uncertainties of the colors and magnitudes of each point in Figure 2?

1A16)
Uncertainties have been added to Figure 2, and their estimation described in Methods as:
The errors on the colour values reflect the spread resulting from using either of the background areas on its own, in addition to the error on the brightness in each band, which is measured by the standard deviation of pixel values.

Q17) I suggest including the pronunciation of the stream name. This has not typically been included in stream discovery publications, which in many cases has led to confusion among the community. I think it's great to name these systems after terms from diverse cultures around the world, but in order to properly respect the cultures and languages the names are borrowed from, it is important that we pronounce the names correctly.

1A17) We thank the referee for the suggestion and agree that pronunciation is important. We have added this to footnote 1.

Q18) In Figure 3, it would help to add the red dots that are shown in Extended Data Fig 3 to the corner plot, since those values correspond to the stream shown in the right-hand panel of that figure.

1A18) We agree and have added these.

Q19) Relatedly, in Extended Data Fig 3, it would be nice to have red lines showing the positions of the selected parameter values in the 1D marginalized histograms.

1A19) We agree and have added these. Note that this is now Extended Data Figure 4.

Q20) There is a typo on line 308: "which where included in the fit"

1A20) We have fixed this.

Q21) There is a typo in Code Availability: "streamsculpture"

1A21) We have fixed this.

Referee #2::

Summary of the key results.
The authors report the discovery of the first extragalactic stellar stream consistent with a tidally stripped globular cluster, identified in deep Hubble Space Telescope imaging of the ultra-diffuse galaxy UGC9050-Dw1. They present HST and CFHT imaging of the galaxy showing a bow-shaped feature next to a bright point source with a signal prominence of 15. The galaxy was first identified and reported in Fielder et al. 2o23 who claimed that most of the GCs likely formed simultaneously during a past dwarf merger event. The authors here suggest several observational lines of evidence supporting the globular-cluster-origin interpretation, including the stream's narrow morphology, coherent structure, colour, surface brightness, and its apparent association with a compact source interpreted as the progenitor cluster.
Using generative modelling of the stream, the authors provide the first stream-based constraint on the dark matter halo of an ultra-diffuse galaxy. Their modelling places limits on the inner density profile of UGC9050-Dw1 and yields dynamical bounds consistent with independent halo mass estimates derived from globular cluster counts. The inferred properties of the stream are also consistent with expectations for a globular cluster progenitor within such a galaxy.
The authors argue that extending stream analyses beyond the Milky Way opens a new avenue for probing dark matter in low-mass, low-surface-brightness galaxies. They emphasize that previously suggested extragalactic globular-cluster–origin streams have either been ruled out or lacked statistically significant detections, making this the first robust example. The work therefore establishes a new method for testing competing models of dark matter by leveraging stellar streams in external galaxies.
The central claim of the paper rests on the identification and interpretation of a very faint structure as a stellar stream associated with point sources in a low surface brightness galaxy at a distance of approximately 35 Mpc, which the authors suggest may be globular clusters belonging to the system. While the authors demonstrate that the faint structure is present in both the HST and CFHT imaging and is therefore unlikely to be a noise artifact, the evidence supporting its interpretation as a stellar stream remains weak. The color–magnitude diagram analysis does not provide convincing support for a globular cluster origin of the point sources, and much of the evidence presented is circumstantial. Alternative explanations, including projection effects or dust-induced features that can mimic similar colors and morphologies, are not sufficiently ruled out.

Although future JWST imaging may offer valuable constraints on the nature of this structure, more rigorous tests using the existing data are required before the paper can

be recommended for Nature. Not enough information is provided on the methodology of the stream detection to judge the validity of the result.

Originality and significance: if not novel, please include references.
The detection and use of stellar streams as probes of dark matter has been extensively developed in the context of the Milky Way, where thin, low–surface-brightness globular-cluster streams have been shown to provide sensitive constraints on the halo potential and small-scale structure. However, identifying such streams in external galaxies has long been considered beyond current capabilities, given their extreme faintness and narrow morphology. Even within the Local Group, previous extragalactic globular-cluster–origin stream candidates have not held up to scrutiny or lacked statistically significant detections above the background. The prevailing view had been that facilities such as Rubin Observatory would be required before such structures could be reliably observed beyond the Milky Way.
In this context, the identification of a compelling extragalactic globular-cluster stellar stream candidate in deep HST imaging represents a significant and novel advance. If confirmed, such streams offer a powerful and currently untapped probe of globular cluster ages and metallicities (thought to trace early stages of galaxy assembly), the accretion history of the host galaxy, and the inner density profile of low-mass dark matter halos. These are topics of central importance to galaxy formation and to distinguishing between competing dark matter models. The ability to extract this level of information from an ultra-diffuse galaxy, using stream-based dynamical modelling, would mark a major step forward and could open a new regime for studying extragalactic halos and age–metallicity relations.

We thank the referee for the feedback and suggestions, which we have considered and answered in detail below.

Data & methodology: validity of approach, quality of data, quality of presentation.
Detection of the stellar stream candidate

Q1) Lines 263–266: In the CFHT data, the putative stream is detected only in the g band and not in the u, r, i, or z bands. While the integration time and seeing are reported for the g-band image, corresponding information for the other filters is not provided. It is therefore unclear whether the g band is the deepest image in the survey or simply has the most favorable image quality.
The authors should clarify whether the g band represents the deepest or highest-resolution CFHT data.

2A1)

We thank the referee for suggesting this opportunity for increased robustness. The g-band image reaches the deepest limiting magnitude (25.14 for a 5 sigma detection of extended objects) of the available CFHT data. The spatial resolution is comparable across all bands, since the observations are seeing-limited.

To enhance the signal, we have stacked the data from the W-3-1-3 and W-3-2-3 fields, which both include the area of interest. This allows us to visually identify the feature in r and i images also. For completeness we have added Extended Data Figure 1 with all the available data in the area of interest, based on your suggestion in Q3.

We have clarified the data selection in the manuscript:
The area containing UGC9050-Dw1 is visible in both the W3-1-3 and W3-3-3 fields of the survey, which contains bands u, g, r, i, and z, so a total of 10 CFHT images are available. Because of the S/N levels we are not able to confidently identify the stream-like feature in the u or z-band images. Integration times, observation depth and seeing for the CFHT data is published in Gwyn et al., (2012).

Q2) Quantitative upper limits on the surface brightness or flux of the feature in the u, r, i, and z bands should be provided. These limits should then be compared to expectations for a stellar stream originating from a globular cluster, based on Milky Way streams, and to the surface brightness measurements derived from the HST data. In addition, it would be helpful to know whether the authors have attempted to identify the feature in stacked or combined u, r, i, and z images.

2A2)

We agree with the referee, and have added the following comparisons to MW streams' surface brightness:
The surface brightness of the stream in the HST images is F555W $=27.0\pm0.1$ mag/arcsec^2, F814W $=26.1\pm0.1$ mag/arcsec^2. This is brighter than the average MW stream \citep{Shipp2018}, which is to be expected for an extragalactic discovery.

Most GC streams known in the MW have lower surface brightness (>30 mag/arcsec^2) (see e.g. Shipp et al, 2018) than this. They are older populations and there is a strong selection bias - we are able to see much fainter things in the MW than in external galaxies.

We show that within selections of progenitor mass and stellar population characteristics it is possible to get surface brightnesses as large as what we measure within that area. We have studied the CFHT data in more depth, and can measure the brightness in the i and r bands too.
Here are the magnitudes of the stream area measured in the CFHT bands:
g W-1-3-1 (W-2-3-2): 25.98 +0.36-0.27 (25.98 +0.30-0.24), lim.magG = 25.14
i W-1-3-1 (W2-3-3): 24.46 +1.49-0.60 (25.29 +0.74-0.44), lim.magG = 24.04 (24.28)
r W-1-3-1 (W-2-3-2): 25.22 +0.40-0.29 (25.42 +0.44 -0.31), lim.magG = 24.51 (24.54)

For all 6 observations, the brightness of the stream area is about 1 mag fainter than the 5-sigma extended object detection threshold (lim.magG). Their prominence is described in our answer to Q4.

Stacking the images across the fields (i.e. stacking the g-image from W3-1-3 and W3-2-3 fields) does enhance it slightly, but when stacking across filters the difference in noise levels and depth dominates, and the resulting image does not show the feature more clearly.

Q3) To allow an independent assessment, image cutouts centered on the candidate stream region should be shown for all five CFHT filters, presented with consistent scaling.

2A3)
We thank the referee for pointing this out. All images are now available in the Supplementary Material.

Prominence of the feature
Q4) The manuscript refers to the prominence of the feature, but it is not clear in which image or filter this quantity is calculated. The authors should specify the exact image used and report the prominence consistently across all available CFHT and HST filters.

2A4)
We have measured the prominence in all filters of the CFHT data, using the amplitude of the fitted gaussian (A/sqrt(A)), and they range from 0.001 in the W3-1-3 u-band to 3.9 in the W3-1-3 g-band (compared to 6.8 in the HST combined image).
The full list is here:
W3-1-3:
g: 3.911
r: 3.010

i: 2.340
u: 0.001
z: 1.767
W3-2-3:
g: 2.962
r: 2.679
i: 3.664
u: 0.004
z: nan (did not converge).

We have added to the manuscript: …*for the HST image, and similar fits to the CFHT images give ratios ranging between 2.3 and 3.9 in all g, r, and i band images, while the feature is not detectable in the u and z bands.*

Q5) Lines 313–314: The mask shown in Extended Data Figure 2 is used for both prominence and color measurements, yet the procedure for constructing this mask is not described in sufficient detail. In particular, the authors should clarify which image is used to define the manually selected points along the track and how sensitive the results are to this choice.

2A5)

The original points selected to fit the central curve of the stream are defined using the HST F814W image. We have outlined the process of creating the masks in the Methods section, adding:
We draw masks around the stream by following a polynomial fit to points selected manually in the F814W image, creating a curved shape with constant width, excluding the GC candidate. We duplicate these on both sides to mask the background areas.

Properties of the stellar stream candidate
Q6) Lines 135–142 and 340–353: The stellar stream is characterized as having a mass approximately 20 times that of Palomar 5, based on stellar population simulations anchored to Pal 5. It is unclear whether alternative stellar populations have been explored. For example, a disrupting young star cluster could plausibly produce a brighter feature with a lower total mass. The authors should test a broader range of stellar population models and discuss how strongly their conclusions depend on the Pal 5 analogy.

2A6)

The most general way to address this is to simply measure which stellar mass it would take for the stream to have the measured surface brightness in the available bands. It still requires choices about the IMF, stellar evolution, and mass loss history, and ignores effects of possible mass segregation in the progenitor cluster. We have used the IMF measured for the Pal-5 cluster remnant, which is a shallow powerlaw (Grillmair 2001), and isochrones calculated using the PARSEC 1.2 model. The IMF mass range is truncated to match the isochrones, which, since significant tidal disruption requires older populations, go from around 0.09 to 1.2 Msol. Anything more massive than that is assumed to have died.

With this, we sample a stellar population and calculate its total luminosity in the relevant observational bands for 30 different isochrones, varying the age from 8 to 12 Gyr, and the metallicity [M/H] from -3 to -0.5. Fielder+ 2023 finds the limits age > 1.5 Gyr and [M/H] < -0.57 for the GC population in UGC9050-Dw1. This age is too low to allow for significant amounts of tidal stripping, so we set the lower bound to a more realistic 8 Gyr.

We vary the total mass of the population sampled, and use an area of 16 arcsec^2 (twice the area of the stream mask used on the data, since we only see one arm) to calculate the surface brightness. We compare to the surface brightness measured in the HST data, which is 27.00 mag/arcsec^2 in F555W and 26.08 in F814W (across the 8 arcsec^2 mask).

The lowest total mass to give surface brightnesses \geq that of the data is 6.2e4 Msol, which is bright enough in the youngest isochrones. We show here an illustration for 7.0e4 Msol, highlighting (with black text) in H814W that several isochrones yield a surface brightness of 26.1 mag/arsec^2 or brighter. All these also yield a F555W surface brightness in agreement with data.

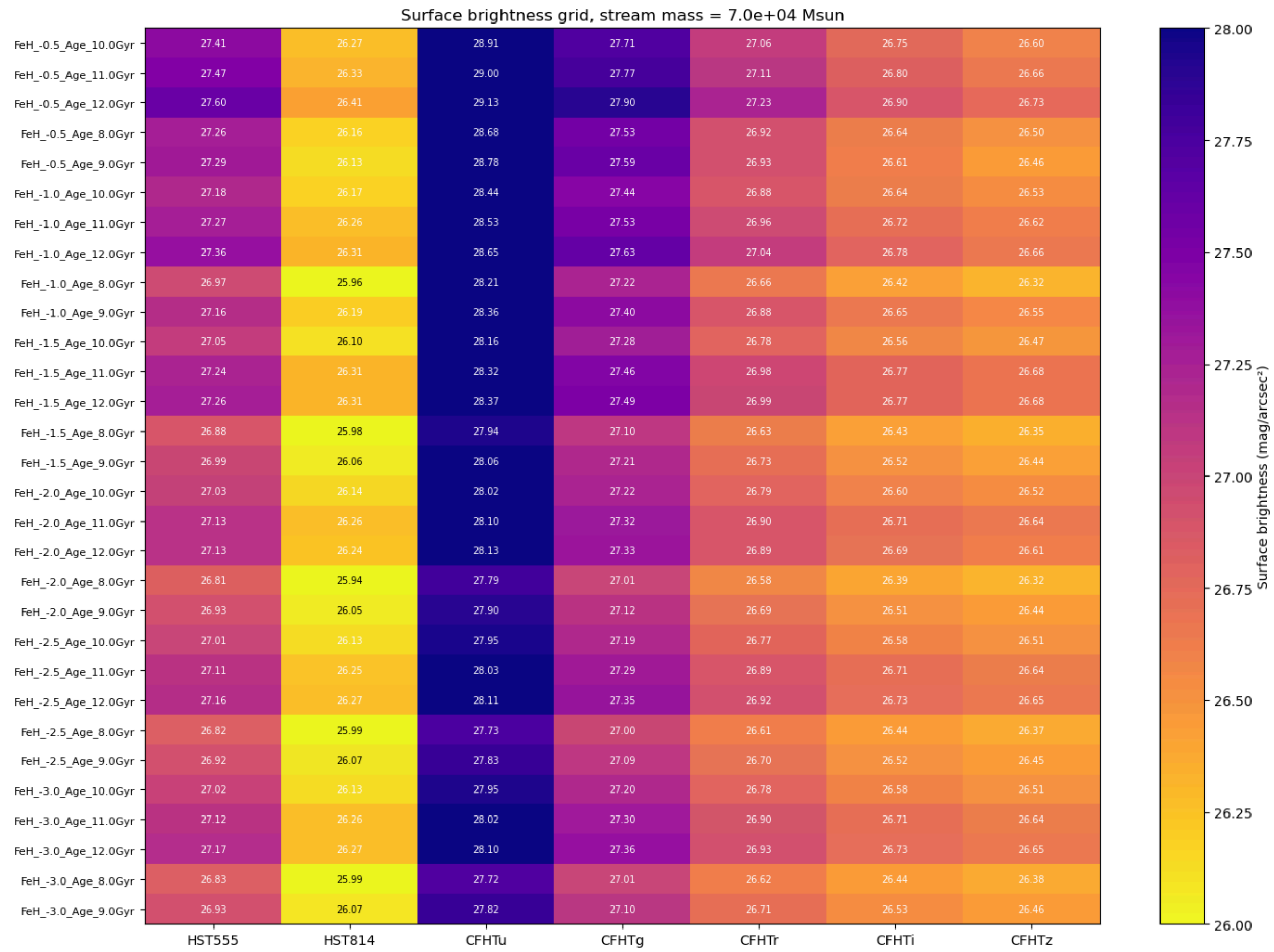

The result of our isochrone scan*. The isochrones on the vertical axis and their brightness in each telescope filter on the horizontal. The color and numbers in each cell is surface brightness over an 16 arcsec^2 area, and black numbers indicate brighter than the observed F814W surface brightness.*

To make that measure meaningful, we need to compare it to values from the literature. For example, Palormar 5, has recent estimates of the total current mass between 1.2 and 9.0e4 Msol (Ibata2017 and Price-Whelan2019), and with a GC remnant mass estimate of only 4300 Msol (Ibata2017) this leaves the stream mass estimate to range from 7.7e3 to 8.6e4 Msol. Our mass estimate for these populations fall within those boundaries. The isochrone needed to get this mass has a different age and metallicity than Palomar 5, but this example serves to show that a stream with a Palomar-5 like mass could produce this feature if the stellar population is a few Gyrs younger.

To relate it to some total progenitor mass, we would need details on mass-loss history or mass ratio between the stream and GC, which are unavailable. By using the assumptions described in A5, we can scale the lower bound stream mass of 6.2e4 M_sun to a total mass of the progenitor system. If we take into account that this is only

one arm, and assume the arms are symmetrical, we use the mass ratio of ~0.75 and the total mass becomes 1.65e5 Msol.

We use this population as our lower bound to what is possible: An isochrone of age 8 Gyr, [M/H]= -1, and total initial progenitor stellar mass 1.65e5 M_sol.

This approach gets rid of the reliance on the Pal5 star count and isochrone choice. It assumes a stellar IMF that is identical in the stream and the GC, and that it is similar to the measured IMF of Pal5. Different IMF descriptions will give varying results, but we find this to be the least arbitrary choice.

We caution that the comparison between the two models we call the lower bound-stream and 20xPal5-like stream is not a direct apples-to-apples comparison. The "lower bound" is sampled until a population of a certain stellar mass is reached, which is bright enough. This total mass is used directly as the stellar mass in the stream arms. The "20xPal5" stream is sampled until a population with a certain *starcount* is reached, which makes it bright enough. The inferred total progenitor mass is found by scaling a separate estimate of Pal5's initial mass to this starcount, which gives that 20x M_pal5 = 2e6M_sun. But the total mass of the stars in the simulated population is significantly smaller than this number, even if scaled by large mass ratios. The total mass of the simulated population does agree within the range of estimated pal5 stream mass of 7.7e3 to 8.6e4 Msol from Ibata2017 and Price-Whelan2019 mentioned above.
These two simulated populations represent two different approaches to estimating the mass, and can be taken as examples of the extremes: The "lower bound" population assumes that every star is still within the measured region, and the "20xPal5" assumes the same mass-loss history as Pal5 and describes the initial mass of the cluster before any disruption occurs.

We have updated this paragraph in Methods and amended the main text to reflect this new approach.

Q7) The possibility that the feature is associated with dust is not adequately addressed. The morphology and colors could also be consistent with a dusty region, particularly given the apparent void immediately below the feature, which could correspond to a region of high attenuation. This is especially relevant if the host system is a dusty, young dwarf galaxy. The authors should test whether dust attenuation could plausibly reproduce the observed morphology and colors..

2A7)

The hypothesis of a dust origin can be partially probed by looking at the colour across the area. By using all the available CFHT bands we can get a better sense of that evolution by measuring the brightness in all bands in background areas at different locations with respect to the stream. In the figure below we have illustrated this color gradient in two different ways: on the left figure we explore how the flux in each band evolves across the different background regions, from furthest above to furthest below. The errors reflect the variance of the background distribution.
The CFHT images are background-subtracted, such that 0 corresponds to the mean background, meaning that several background samples have “negative” flux.
The HST observations we have normalised and arbitrarily scaled (*20-20) to fit in a comparable frame, since we only aim to compare trends here.

In the right figure we switch them to illustrate how the flux in each background region varies across bands. The darker the color, the further from the stream. Here the errors are omitted to avoid clutter.
As can be seen in the figure below, there is no discernible trend of colour dependence on distance from the stream, or difference in brightness evolution for the different bands. If anything, all the backgrounds below the feature are less bright in the most red band, which aligns with expectations as they lie closer to the bluer centre of the galaxy. This indicates that it is not a dusty region creating this feature.
The area containing the feature is consistently brighter across all bands.

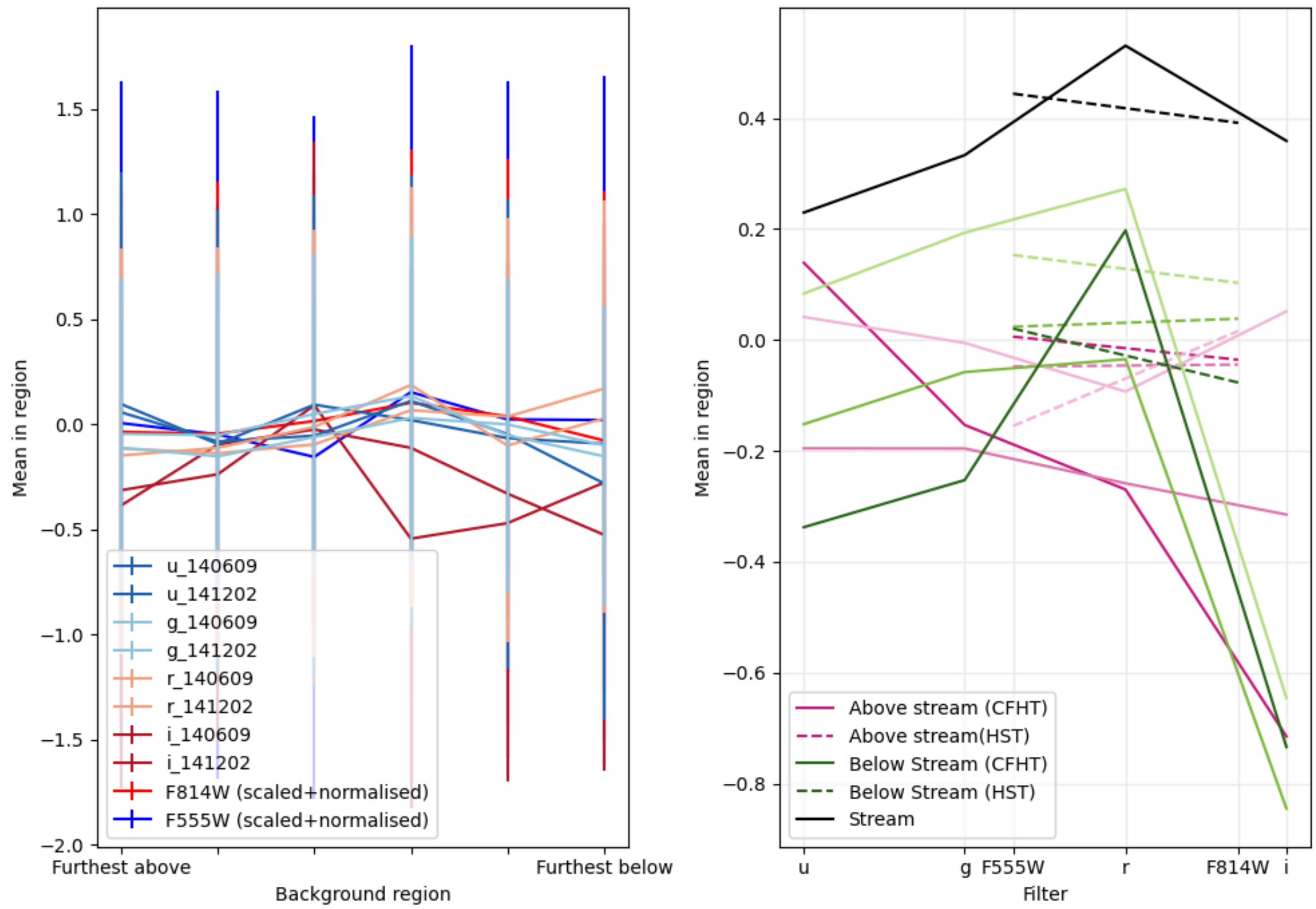

We have added to our discussion:

Alternatively, a dusty region may give rise to fluctuations mimicking the stream candidate. If there were a dust patch in the region on one side of the stream we would expect it to be redder than on the other side. We find no evidence of such a trend.

Color and CMD analysis

Q8) Figure 2: It is unclear what conclusion the color analysis of the stream candidate is intended to support. The comparison is limited to the progenitor candidate within the same galaxy, which does not provide sufficient external context. For comparison, the authors should include color–magnitude diagrams of well-studied stellar streams in the nearby universe. For example, Bonaca et al. (2012) present CMDs of Milky Way globular cluster streams with comparable widths.

Similarly, the globular cluster candidate selection box from Fielder et al. (2023) should be complemented with comparison samples of Milky Way globular clusters, young star clusters in the Milky Way, and clusters in nearby galaxies. Without these comparisons, it is difficult to assess how diagnostic the presented CMD is.

2A8)

We thank the referee for the suggestion, and have added points to the CMD representing MW GCs for comparison. A single color measurement for a GC allows for a range of stellar populations, and the CMD presented here is as such not intended to be diagnostic or tightly constrain the properties of the population. The intend is rather to show that both objects fall within the CMD selection criteria of Fielder et al., and that their colors are consistent with each other.

Q9) More broadly, the analysis relies on a single color–magnitude comparison. Given the availability of multi-band data, the authors should demonstrate whether additional colors show consistent localization. Recent work, such as arXiv:2306.06164, illustrates the power of multi-color constraints in establishing stellar population coherence. The authors should also quantify how much age and metallicity variation is permitted within the observed color scatter, and whether the test shown is sufficient to support the conclusion of a common stellar population.

2A9)

Because of the lower resolution and larger S/N, the CFHT colors are inadequate for a detailed colour analysis. We show here the CMD with the g-i colors from CFHT filters to illustrate that the errors span almost the entire region of interest. Since CFHT g is significantly different from Johnson V, the actual values should not be considered accurate, and we show them here only to illustrate the large errors.

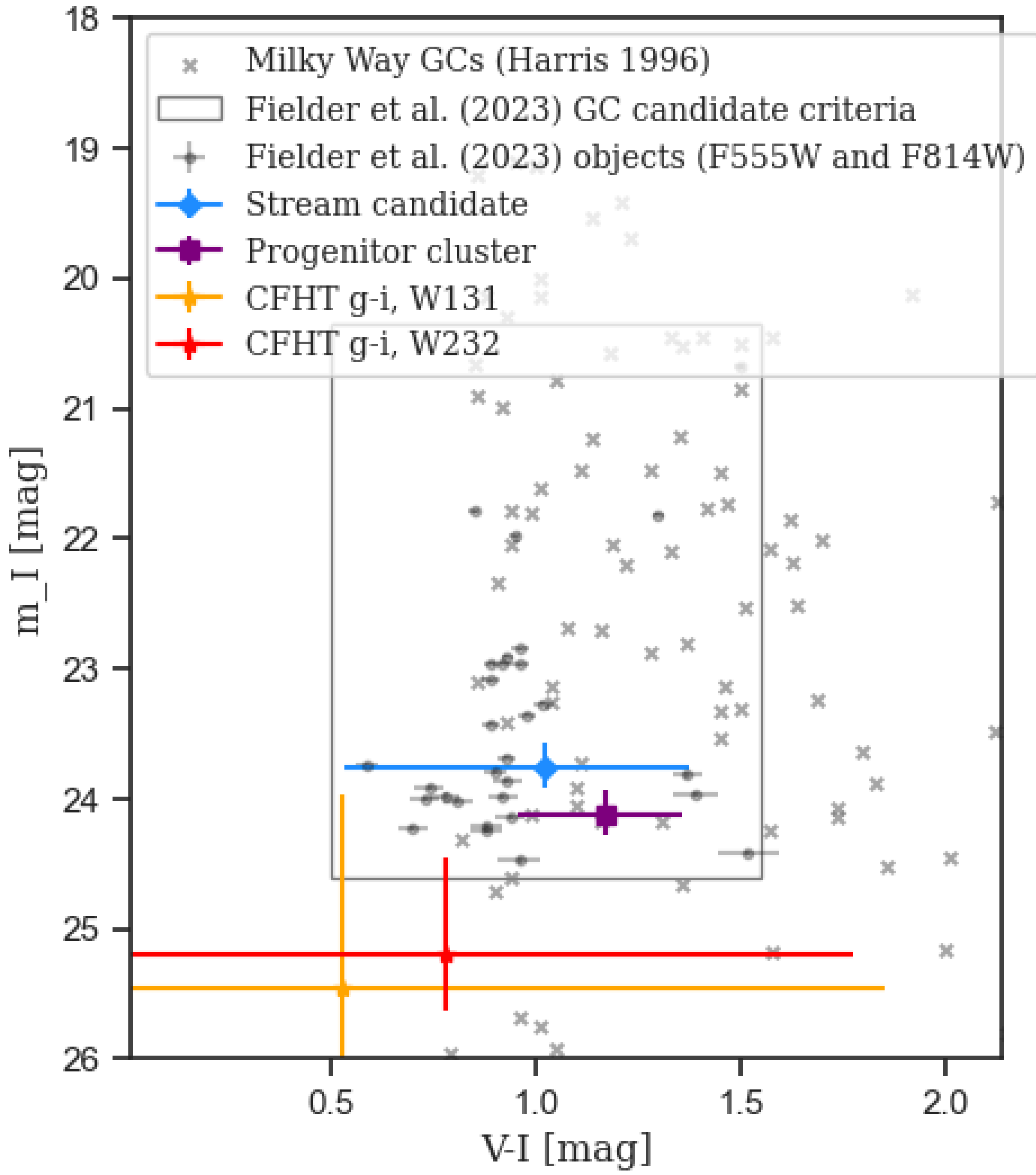

To explore the color dependence on the stellar population, we have created a CMD based on all the isochrones from our scan described in A6. This shows a range of F555W - F814W between 0.8 and 1.2, and thus all fall within the grey selection box. This supports the conclusion of Fielder et al. 2023 that the single colors of the GC candidates can only place mild lower (upper) bounds on the age (metallicity) of the general population. However, we refer to the comparison to other known MW clusters, which are shown as crosses in the plot above, that many of those are substantially redder than this range.

Q10) If reddening is present, this further complicates the interpretation. The authors should explicitly address the possibility of internal or foreground reddening and discuss how it affects their stellar population inferences.

2A10)

The UDG lies in a direction with a low degree of foreground reddening, with a correction of only 0.055 and 0.031 mag in the two HST bands. The physical width scale (<1 arcsec) of the MW dust filament required to make this feature is also far below anything previously detected (see e.g. Juvela & Mannfors, 2023). If dust plays a role, it is in all likelihood in the host galaxy. If variation of dust should create this feature, we would expect a gradient across the feature. In the HI data (VLA and Apeterif) presented in Fielder+23, they show isodensity contours going orthogonally across the feature, indicating that at least gas fluctuations are not aligned with the stream.
We have performed an additional analysis of the color gradient across the background surrounding the stream. See answer A7) for description of this analysis.

Q11) Association with the host galaxy and signal-to-noise
Several key assumptions remain insufficiently justified. The stream identification appears to rely largely on visual inspection, and it is unclear how objective the shape determination is. The quoted signal-to-noise ratio of 15 requires further justification. Given the lack of a priori evidence that all segments of the feature belong to the same stellar population, the signal-to-noise should be evaluated region by region along the stream.

2A11a)

We thank the referee for the suggestion. To support the manual identification we have used the Rolling Hough Transform (RHT) (Clark et al. 2014) to test if a machine vision algorithm would find the feature. The RHT searches for continuous structure within a moving window, and will find both clumps of light and long streaks. We downsampled the F814W HST image to improve performance and speed, and tested a variety of different hyperparameters for the search, while keeping the threshold for detection high. Consistently, the HRT finds the curved structure. In many cases it finds other stuff as well, but few are consistent across images except our stream candidate. A sample of these runs are shown in the figure below. Notice especially the top right corner, which is the lowest amount of downsampling and the highest parameter scales, and it finds the central stream track neatly without flagging much else that might be of interest.
Below is shown the F814W image and the HRT tracks on top (in red/yellow), for a sample of 12 different parameter choices.
d refers to the amount of downsampling (e.g. d5 means 5 pixels combined into 1) and *s* is the Gaussian smoothing kernel performed on the image before the HRT.
wlen is the window length of the RHT, *smr* is the smoothing radius used to remove background in RHT, and *thres* is the RHT threshold for accepting association.
The manually drawn stream track, which is used in our analyses, is illustrated by a white oultine spanning plus and minus 0.0008 degrees in RA.

As illustrated in the figure below, the Rolling Hough Transform consistently identifies both the position and curvature of the feature. We have included this in the manuscript by adding:

The feature was initially detected by eye, but applying the Rolling Hough Transform (Clark et al., 2014) with a range of (hyper)parameters consistently returns both the location and curvature of the structure. We use this as a validation of the initial by-eye detection.

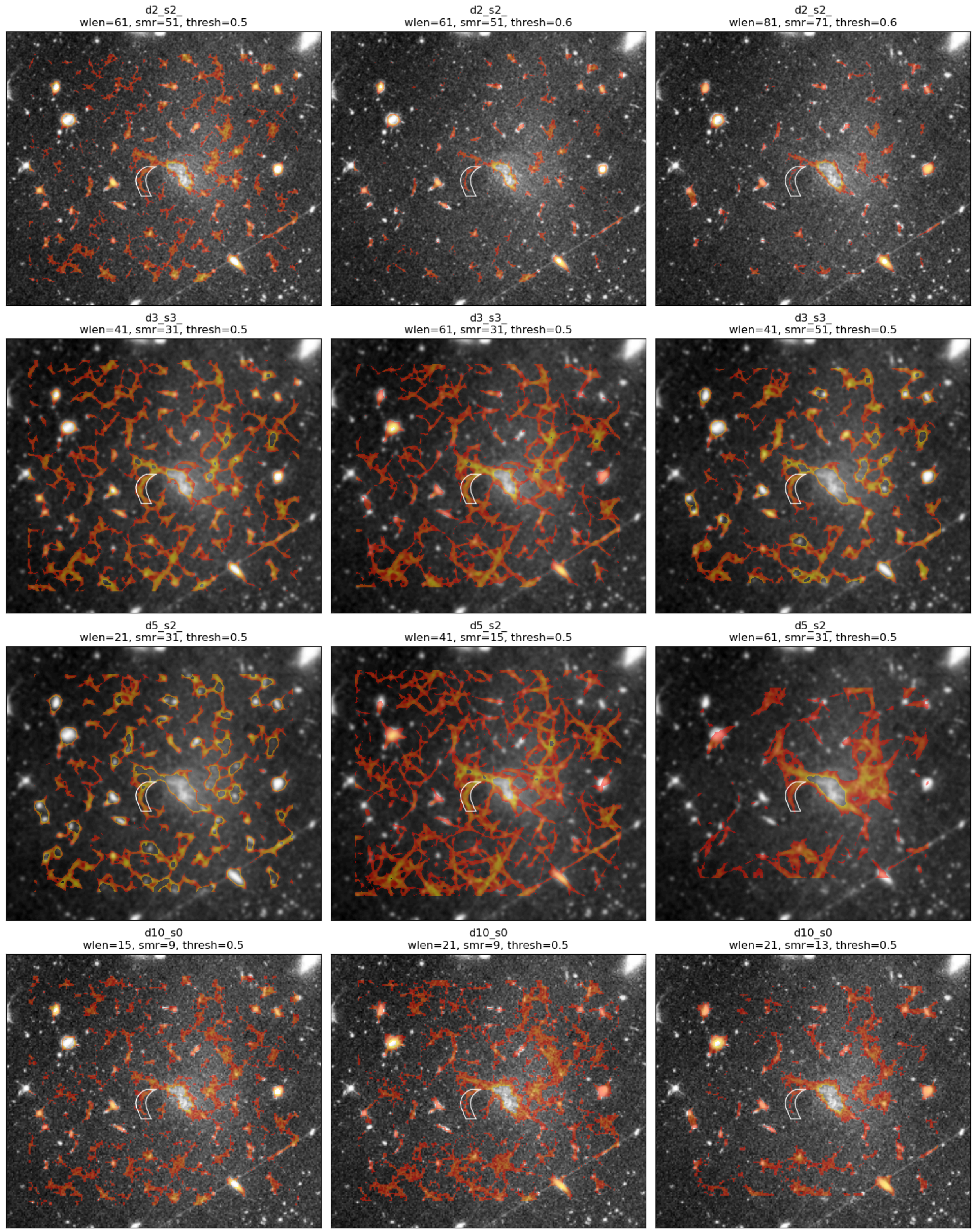

The Rolling Hough Transform *(red-yellow) consistently finds the stream candidate position and curvature (white outline) in the F814W image.*

2A11b)

In terms of the signal significance we appreciate the reviewer highlighting a lack of clarity. The statistical significance contains three main components: the probability that it is a stream, the probability that we would find it, and, given the first two statements are true, the statistical significance of the signal in the data. What we quote as a significance is the latter. The probability of it being a stream we address qualitatively by our combined analyses, and the probability of detecting such a signal given it exists somewhere in an image is not dealt with. It is common practice in many fields of astrophysics to rely partly on our eyes' ability to detect patterns and features in images. In the case of this feature, it was noticed in Fielder et al. 2023 serendipitously by one of the authors, who was not looking for streams or features like it, as well as by the RHT algorithm as described above. A complete statistical exploration accounting for trials factors, look-elsewhere, stochasticity, selection bias, the frequency of extragalactic GC streams and the variability in their appearance, etc., is beyond the scope of this paper. One common feature of stellar streams, especially those of GC origin, is that each small segment of the stream is, with only photometric data, rarely bright enough to be noticeable, but rather the continuous, linear and slightly curved structure is what makes a stream stand out.

2A11c)

We have repeated the amplitude-based significance (A/sqrt(sigma_A) on segments through the feature, and the results can be seen below. For this, we included the GC to demonstrate how it would likely dominate the fit if it were included in the global fit. The black dashed line is the fit to total stream (without GC) that is quoted in the paper, which is included for comparison. All but one of the segments have a fit that converges to a similar Gaussian as the global fit, which indicates that no one segments dominates the fit. The significance is of course lower for each of them, since there is less data in each segment, so the poisson star-count noise is relatively larger, and the best-fit amplitude is less certain. The fit parameters are allowed to vary freely, except the amplitude and spread which are limited to positive values. The initial values are decided automatically and individually based on the mean, standard deviation and min/max values of the data being fitted to. The exact fit parameters naturally depend on the segment placement and size, which is why we continue to use the global fit values for all subsequent analyses.
In the right panels we have illustrated the area included in the segment. The image has been recast to reflect the perpendicular sampling used to get the means parallel to the stream, so the stream here goes straight along the horizontal center of the image, and the GC can be seen in the right side.

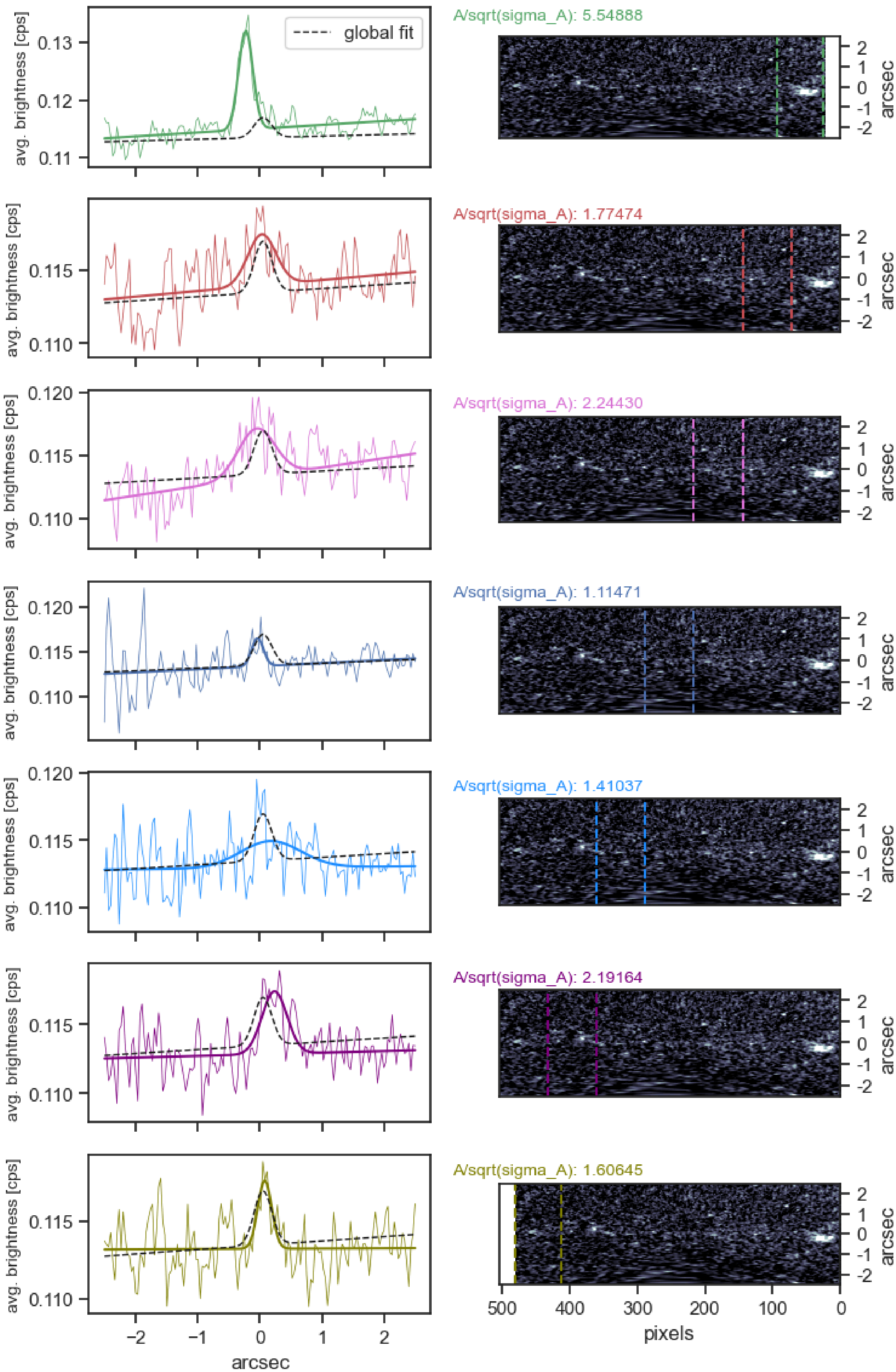

global fit
A/sqrt(sigma_A): 5.54888
A/sqrt(sigma_A): 1.77474
A/sqrt(sigma_A): 2.24430
A/sqrt(sigma_A): 1.11471
A/sqrt(sigma_A): 1.41037
A/sqrt(sigma_A): 2.19164
A/sqrt(sigma_A): 1.60645
avg. brightness [cps]
arcsec
pixels

Q12) It is also not clear how the globular cluster candidates and the stream are confirmed to be physically associated with the ultra-diffuse galaxy rather than being chance projections. The manuscript should more rigorously address this point.

2A12)

We agree with the referee that this should be mentioned as a possible caveat. We have added the following sentence where we we discuss chance alignment in the manuscript:

Lastly, there is the possibility of a chance alignment of unresolved stars in the host galaxy **{\bf or a chance projection of stars unassociated with the host}**.

While this cannot be ruled out, the combined analysis of the SB, dynamical model (length, width, curvature towards center of mass, inferred prog and halo mass), make us confident that this is indeed associated.

Q13) Modeling and statistical robustness

For Figure 3, the authors present a single realization of the modeled stream. It would be more informative to show multiple samples from the posterior distribution to illustrate where the spatial constraints are tight and where they are uncertain. The observational data should also be marked more clearly on these figures.

2A13)

We agree and have added another example of a good fit to Fig 3, as we, since submission, have calculated that for a GC with different age and metallicity than Pal 5, a stream with an even lower mass progenitor could be observable in the data. We have added the respective red points and lines in the corner plot to reflect the stream models visualized (as suggested by referee 1). We have also made the observed data clearer on all model figures and added them to the legend for figure clarity, thanks for pointing this out.

We have also added an appendix figure (Exteded Data Figure 5) showing five other consistent fits (within 1sigma in in the posterior) and five inconsistent fits (>2 sigma outliers in the posterior). We discuss in the text how the consistent fits produce the correct curvature, overlap with all of the observed data, is not too diffuse and produce streams with shorter lengths within a reasonable halo mass range.

Q14) The modeling does not appear to account for surface brightness fluctuations or clumpiness along the stream. The authors should discuss whether such effects could bias the inferred morphology or width.

2A14)

We agree that particle-spray type simulations do not capture the detailed density of streams and their substructure. However, X-Stream relies on control points (not density), and we apply an inverse density weighing to smooth over features. We also test different mask widths in the paper and refer the referee to the new observational tests analysing the stream width in segments which is shown in A11. This highlights that no single segment is dominating the width measurement. An offset object near the end of the stream (purple segment in A11) can increase the measured width slightly. As we briefly mention in Methods, if the stream track is clipped before that point the width decreases, but is still within expectation from a GC stream. We note that streams generally become more diffuse further from the progenitor.

To address the referees comment, we have added a new sentence to the methods generative stream models section:

{\bf Note that particle-spray models do not reproduce the detailed density along the stream, but X-Stream relies on control points and includes an inverse density weighing to smooth over features.}

Q15) Statements such as “we find that a GC stream origin of the observed feature is dynamically plausible” would benefit from a quantitative assessment of confidence. Is it possible to place statistical bounds on this plausibility, rather than relying on qualitative agreement?

2A15)

We agree with the referee that this statement is too vague and have instead reminded the reader in the manuscript of the quantitative upper bound on the progenitor mass.

Please also see our new mock observations, where we show that a dwarf stream would be observed as wider (Extended data figure 6), and our new discussion of why wider streams are disfavored by the sampler (Extended data figure 5). Combined, we think that these new analyses have added more clarity and a more quantitative assessment of confidence.

Q16) Similarly, the claim that the perpendicular brightness profile resembles a stream more than a shell would be strengthened by explicitly showing both profiles in one-dimensional plots for direct comparison.

2A16)

This is certainly a point worth clarifying. While indeed there is no hint of the asymmetric shape that would be clearly indicative of a shell, detecting that asymmetry relies on observing even lower surface brightness components inside the shell caustic that may not be visible here. Therefore the profile is not suggestive of a shell but not dispositive either.

Particle-spray methods like those that underlie X-Stream do not make particularly realistic shells as the constant-rate Lagrange-point loss approximation breaks down, so we have put together the following comparisons with a simple test-particle mode. We assume the fitted halo mass and progenitor position from the paper but on a much lower angular momentum (more radial) orbit. For both the “Lower Bound” model at the top and the “20x Pal5” at bottom, there is some density cut above which both stream and shell would look primarily symmetrical.

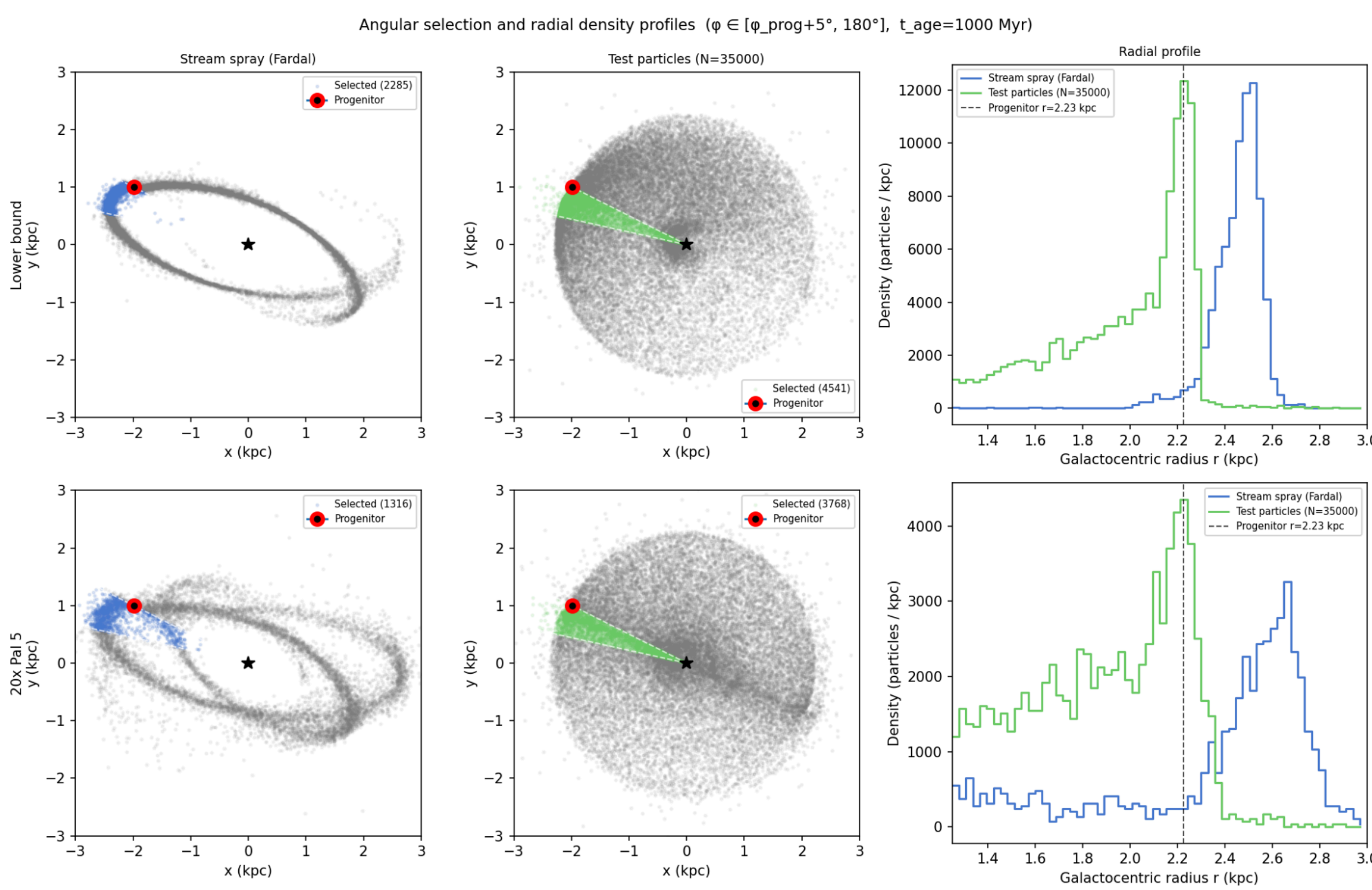

We show the effect here in mock images; the surface brightness scaling is arbitrary but the concept that the asymmetry fades with detection significance is clear.

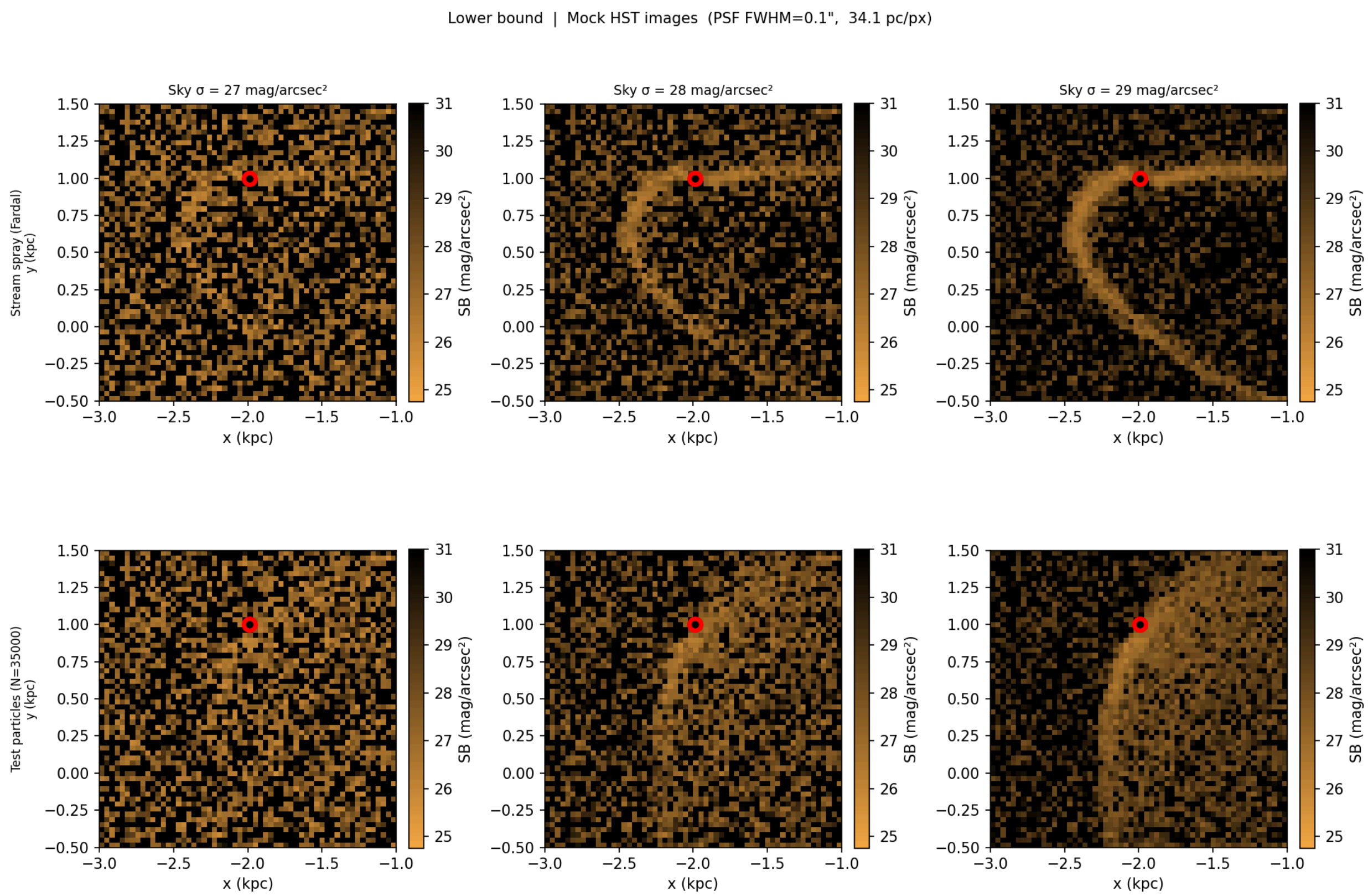

These images also display some better evidence that the observed object is more likely a stream than a shell: its curvature. Streams are seen in some projection; especially if only one tail is observed, they can have a variety of orientations and curvatures (Nibauer et al. 2023). . Shells by contrast *are themselves* projection effects caused by stars accumulating at the apocenters of their orbits and so approximate isopotentials. They should have a center of curvature closely matching the center of their host. The stream candidate’s radius of curvature is far too tight and its center of curvature is skewed at a substantial angle, approximately 30 degrees, from the center of UGC9050-Dw1.

Below this concept is applied to a few cases from the literature and this work. In all cases the marks are done by eye for illustration purposes only.

NGC 474 from Duc et al. 2015

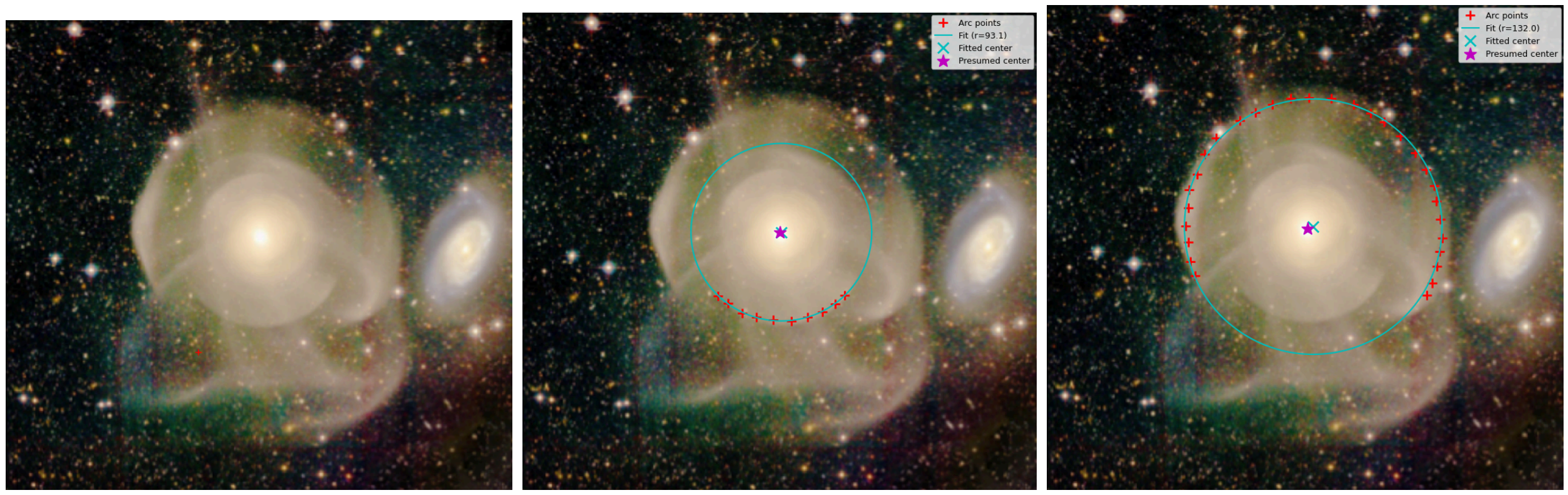

Simulation from Hendel 2015

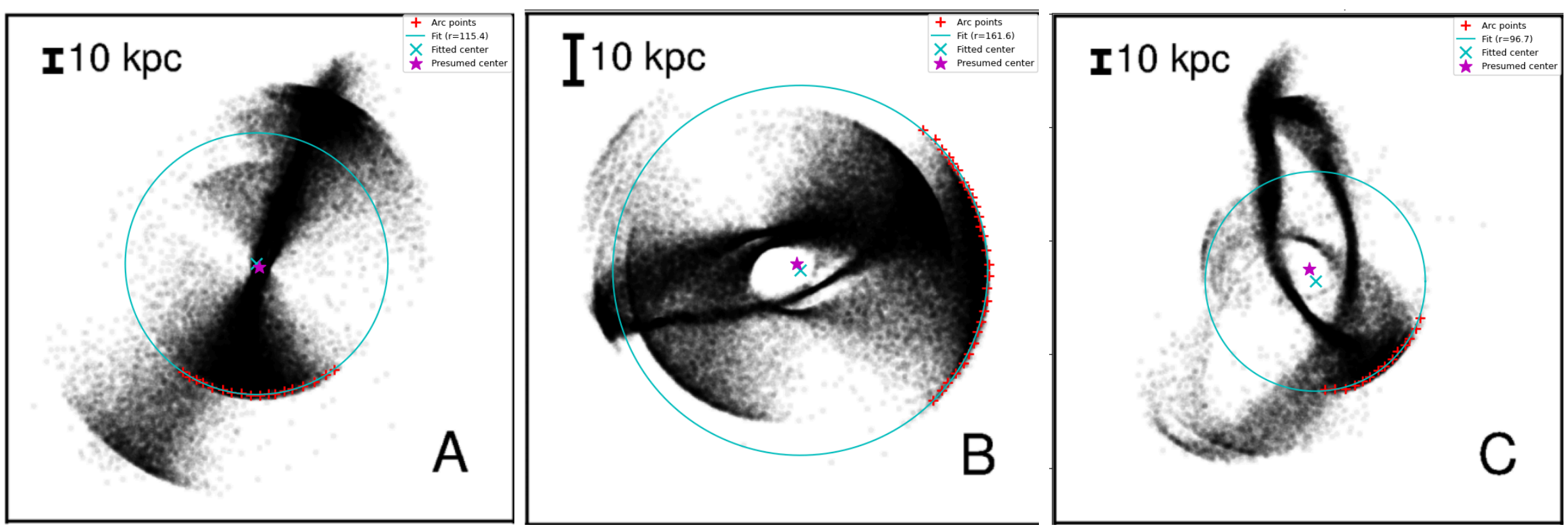

This work

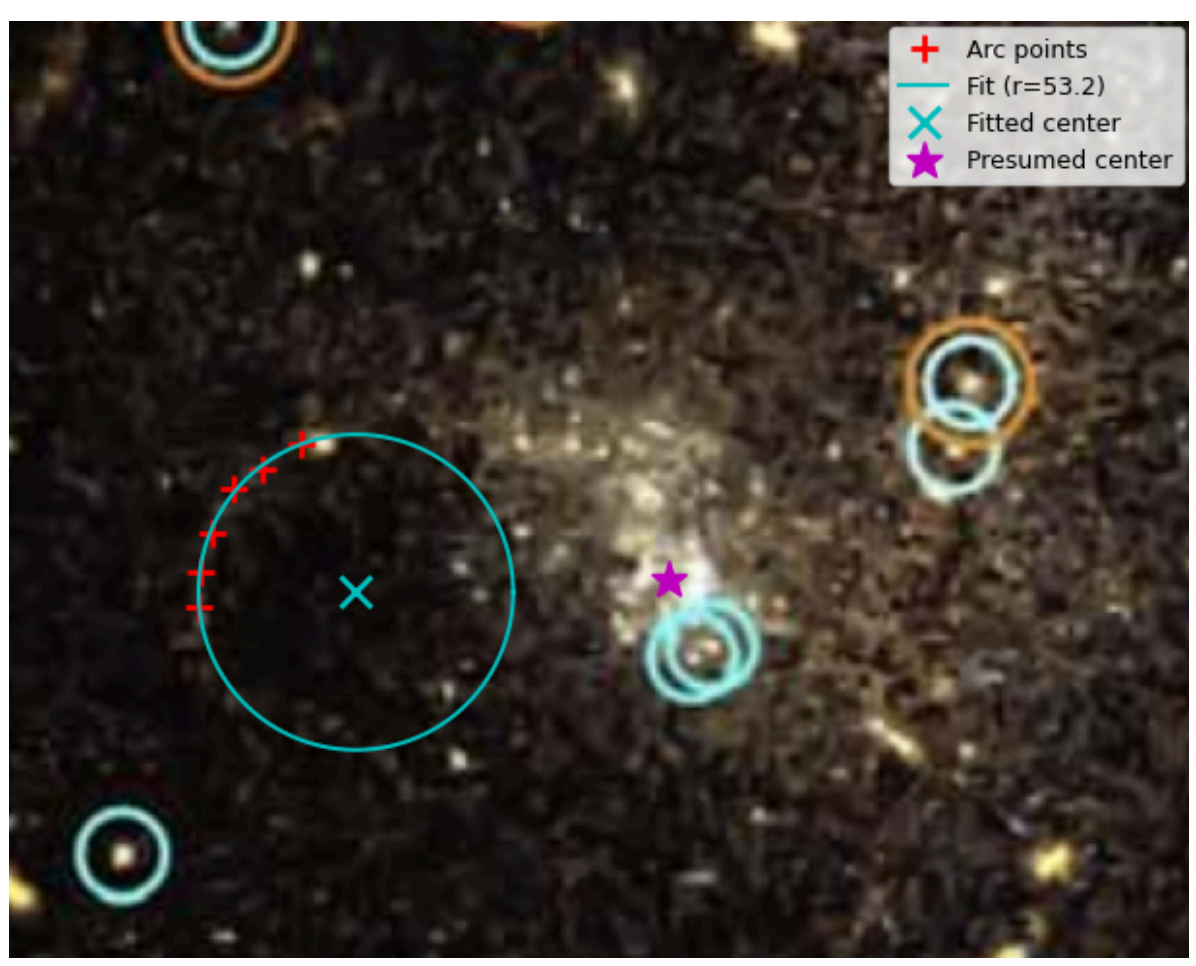

This seems too far of an excursion for the main body of the text but we have added this as supporting evidence to the shell commentary section.

Q17) Finally, Figure 1 does not clearly highlight the feature of interest. For comparison, Figure 1 of Fielder et al. (2023) provides a much clearer visualization of the relevant

structure. Improving the presentation of the discovery image would significantly aid the reader.

2A17)

The stacking and scaling applied to the original Figure 1 was not optimal for this purpose, and the exact smoothing or rasterisation algorithm used by Fielder et al. is different, and slightly highlights the feature by coincidence.
We have updated the appearance of Figure 1 to resemble that of Fielder et al. more, and hope that this increases the visibility of the stream to the reader.

Q18) Appropriate use of statistics and treatment of uncertainties.
The color–magnitude diagram on Fig 2 lack y-error bars. Do Fielder+23 report error for their CMD?

2A18)

We thank the referee for noticing this oversight. Fielder et al. provide errors on the magnitude for the objects inside the selection box, in the F555W and F814W bands (while their CMD is in V-I, they do not provide errors in those bands) and these are shown in our response to question 9).
In the manuscript we have kept the errorbars off these points to avoid confusion, since they are only available for part of the sample and not in the filters used for the CMD. We have added y-errorbars for the stream and GC progenitor.

Q19) Several key results appear to rely on visually selected regions or manually defined masks, yet the sensitivity of the results to these choices is not explored.

2A19)

In response to the above suggestions we have validated our by-eye detection with a machine vision algorithm (see answer to Q11), we have tested different stellar populations, mock observed streams to ensure that they would look as expected. We have run our numerical simulations with masks of different widths, and clarified how X-Stream treats wider and/or longer streams.

Conclusions: robustness, validity, reliability.
Please see the above sections.
Suggested improvements: experiments, data for possible revision

Q20) If possible, the authors should analyze the candidate stream in spatially distinct segments rather than treating it as a single structure. The signal-to-noise ratio should be

evaluated independently along different portions of the feature to assess whether the detection is robust throughout. Photometry from these segments should then be added to the CMD analysis to test whether they are all consistent with a common stellar population within uncertainties. This would provide a more statistically grounded assessment of whether the feature represents a coherent stellar stream.

2A20)

We have repeated the signal significance and color measurements in segments along the feature. Choosing the number of segments is rather arbitrary, and using more segments results in decreasing the signal prominence.
See answer A11) for this segment-wise analysis. We have performed the colour measurement in each segment, and while the errors are too large to say anything concrete, 5/7 of the segments' mean colours fall within the selection box in the CMD.

Referee #3:

I co-reviewed this manuscript with one of the reviewers who provided the listed reports.

3A)

We thank the referee for their inputs and refer to responses to the relevant reports.

Referee #4:

I read with interest the paper "Evidence for the First Globular Cluster Stellar Stream beyond the Milky Way" by Kiel Holm and collaborators. The manuscript presents the detection of a possible globular cluster stream in an ultra-diffuse dwarf galaxy UDG9050-Dw1 and uses these data to study the dark matter properties of the host.

The article is interesting and well written, but I have three main issues with it.

Q1a) First, the authors claim that theirs is the first detection of a globular cluster stream beyond the Milky Way. However, quite recently Poulain et al. 2025 (Nature 640, 902) showed clear evidence of stellar streams associated with merging nuclear star clusters in a sample of dwarf galaxies studied with HST imaging.

4A1a)

We discuss this briefly in the manuscript already, but we agree that this deserves clarification in wording. Our main point is that Poulain et al. 2025 reported the discovery of *tidal tails* induced by the merger of two GCs/nuclear star clusters (mutual stripping) rather than GC *stellar streams* which form due to tidal fields from the host leading to lagrange point stripping.

We have clarified this in the submitted manuscript by writing:
“Recent observations have reported evidence of tidal tails from mergers of GCs or nuclear star clusters in dwarf galaxies \citep{Poulain2025}. Tidal tails are formed due to mutual interactions between two low mass ratio objects rather than from Lagrange point stripping of high mass ratio objects such as a GC by a host galaxy, which leads to the formation of stellar streams. In the case of merger induced tails, we expect them to occur near the host centre. This scenario is unlikely to explain the feature presented in this paper, which has a distance $>$2 kpc from the luminosity centre of the host UDG. “

Q1b) Furthermore, in the nearby Andromeda galaxy, stellar streams have been known to be associated with star clusters for many years. Indeed, Pearson et al 2022 (ApJ

926, 166; led by a co-author of this paper, and whose secondary title in the arXiv version of that paper is "GC streams in M31") present a gallery of GC stream candidates. So I am somewhat baffled by the main claim that underlies the present manuscript.

4A1b)

Note that the findings of Pearson et al. 2022a were inconclusive. The authors write in their manuscript that “we could not make conclusive statements regarding their nature.” and “we did not find clear evidence (e.g., as compared to the synthetic streams in Section 4.4) of GC stream in the PAndAS data with a systematic search using the HSS”. Note also that none of the Pearson et al. 2022a candidates had lengths extending beyond one detection patch nor any curvature, despite the prediction of these features in Pearson et al. 2022a Fig 9.

We agree that Pearson et al. 2022 did do a systematic search and found some candidates, but without clear evidence. We point the referee to our submitted manuscript line 78, where we stated that GC stellar streams were “simply not detected at a statistically significant level above the background stellar halo, even in nearby galaxies such as M31 \citep{Pearson2022a}.”.

Q2) Second, the paper uses a method to analyse the properties of the host galaxy which has not yet been refereed. This makes it very difficult to judge the validity of the claims without a thorough examination of that software. My intuition from long experience of modelling stellar streams is that the algorithm cannot possibly work as well as is claimed, since the observed structure is (at best) extremely noisy, no kinematic information is available, and it is only seen in projection. I strongly suspect that the authors are being misled by a perfect match of their model and artificial test data.

4A2)

The Nibauer & Pearson 2025 manuscript has been accepted for publication following minor revisions from the referee. The only substantial change to Nibauer & Pearson in the referee stage, was how they draw contours for their posteriors. Since they sample a negative KL-divergence, the density of samples can be different from the true posterior and can suppress sharp peaks in the probability of a model stream. To correct for this, posterior constraints are now derived by constructing bins in parameter space and taking the average weight of the samples in each bin. Contours are then drawn using the average weight in each bin. This way, the weights inform the drawing of contours rather than the sampling density of the negative KL-divergence. We have updated all plots in this submitted manuscript to reflect this change. The only difference is that the

mprog is now an upper limit rather than a constraint (see Figure 3 and extended data Figure 4), and that some of the quoted 68/95% confidence regions are slightly wider.

We agree with the referee that extragalactic stream modeling using only projected 2D morphology is challenging and that degeneracies exist (see Nibauer & Pearson Fig 7). Note that we do not argue that X-stream can find exact solutions for orbits, progenitor/halo masses etc., we instead argue that X-stream *can rule out* regions parameter space which lead to streams that do not match the input morphological data. Note that we use a wide set of priors allowing for halo masses and progenitor masses spanning several orders of magnitudes and that the minimum length requirement, combined with a width sets limits (see Nibauer and Pearson sec. 4.1). We have added the posteriors of our fiducial run to Extended Data Table 1 to make our findings clearer.

To further address the referee's concern and make our points above clearer in the manuscript, we have added a new figure to the appendix to demonstrate inconsistent stream models (>2sigma outliers, Extended Data Fig 5). These inconsistent fits are bad for different reasons: some don't match the curvature of the input data (e.g. they are too straight or only partially overlap with the input data), some are too long, wide, and diffuse, while others are misaligned with the input data entirely. We have added the following text to the methods section to clarify this:

{\bf Streams wider than the data have excess density inconsistent with the control points, thereby disfavouring wider models.}

Note also that Chemaly et al. 2026 independently finds it possible to constrain halo and progenitor parameters from extragalactic stream imaging and reproduce the general findings of Nibauer & Pearson.

We emphasize that in our submitted manuscript we also allow for wider stream masks, and that we now also include mock observations, where we include a dwarf galaxy-like stream (see the new extended data figure 6 and related manuscript text). All our tests indicate a GC stream.

Q3) Third, I find the images presented here far from convincing, and far from the claimed 6-sigma detection level. In ED Fig 1 one can see how noisy the image is, and a comparison to Fig 1 highlights that one can identify several linear features by eye over a

large field, but which are probably suprious noise alignments. The structure presented in this article looks (to me) possibly real, but I suspect the 6-sigma claim almost certainly ignores the look elsewhere effect.

4A3)
The stacking and scaling applied to the original Figure 1 was not optimal.
We have updated the appearance of Figure 1, and hope that this increases the visibility of the stream to the reader.

In addition to the visual detection we have run the machine vision algorithm Rolling Hough Transform (RHT, Clarke et al, 2012) on the F814W with a suite of different hyper-parameters, and it consistently returns both the location and curvature of the stream, while other features very across the initialisations. For details on this, see our response to the previous referee's Q11a.

In terms of the signal significance we appreciate the reviewer highlighting a lack of clarity. The statistical significance contains three main components: the probability that it is a stream, the probability that we would find it, and, given the first two statements are true, the statistical significance of the signal in the data. What we quote as a significance is the latter. The probability of it being a stream we address qualitatively by our combined analyses, and the probability of detecting such a signal given it exists somewhere in an image is not dealt with. It is common practice in many fields of astrophysics to rely partly on our eyes' ability to detect patterns and features in images. In the case of this feature, it was noticed in Fielder et al. 2023 serendipitously by one of the authors, who was not looking for streams or features like it, and it was now also detected by the RHT algorithm (Clark et al. 2014) as described above. A complete statistical exploration accounting for trials factors, look-elsewhere, stochasticity, selection bias, the frequency of extragalactic GC streams and the variability in their appearance, etc., is beyond the scope of this paper.

We underline that we do not claim a 6 sigma detection certainty. This quoted value is simply a measure of the prominence of the feature in the data compared to the background. We have further clarified our description of how this is done in Methods.

Q4) My conclusion is that the paper is interesting, and presents a glimpse into exciting future science, but the authors' case is not watertight, and the community would benefit from the claims being substantially toned down.

4A4)

We thank the referee. We have added more quantitative assessments throughout the manuscript (see particularly mock observations of a dwarf stream in Extended Data Figure 6, more discussion of why certain fits are ruled out in relation to Extended Data Figure 5).

We have clarified our statistical claim, underlining that we do not claim a discovery with 6 sigma certainty, and that the significance measure refers only to the signal strength in the data. There are many other statistical effects, such as the look-elsewhere effect, which should be included in the complete statistical treatment needed to give an accurate detection significance.
